# New Perspective for Using Antimicrobial and Cell-Penetrating Peptides to Increase Efficacy of Antineoplastic 5-FU in Cancer Cells

**DOI:** 10.3390/jfb14120565

**Published:** 2023-12-12

**Authors:** Nuno Vale, Eduarda Ribeiro, Inês Cruz, Valentina Stulberg, Beate Koksch, Bárbara Costa

**Affiliations:** 1 PerMed Research Group, Center for Health Technology and Services Research (CINTESIS), Rua Doutor Plácido da Costa, 4200-450 Porto, Portugal; eduardaprr@gmail.com (E.R.); inesiocruz8@gmail.com (I.C.); b.c.211297@gmail.com (B.C.); 2 CINTESIS@RISE, Faculty of Medicine, University of Porto, Alameda Hernâni Monteiro, 4200-319 Porto, Portugal; 3 Department of Community Medicine, Health Information and Decision (MEDCIDS), Faculty of Medicine, University of Porto, Rua Doutor Plácido da Costa, 4200-450 Porto, Portugal; 4 ICBAS—School of Medicine and Biomedical Sciences, University of Porto, Rua Jorge Viterbo Ferreira, 228, 4050-313 Porto, Portugal; 5 Institute of Chemistry and Biochemistry, Freie Universität Berlin, Arnimallee 20, 14195 Berlin, Germany; valentina.stulberg@fu-berlin.de (V.S.); beate.koksch@fu-berlin.de (B.K.)

**Keywords:** antimicrobial peptides (AMPs), cell-penetrating peptides (CPPs), anticancer agents, 5-FU, drug delivery, ADMET properties, drug metabolism, combination therapy

## Abstract

This study explores the effectiveness of the antineoplastic agent 5-FU in cancer cells by leveraging the unique properties of cationic antimicrobial peptides (CAMPs) and cell-penetrating peptides (CPPs). Traditional anticancer therapies face substantial limitations, including unfavorable pharmacokinetic profiles and inadequate specificity for tumor sites. These drawbacks often necessitate higher therapeutic agent doses, leading to severe toxicity in normal cells and adverse side effects. Peptides have emerged as promising carriers for targeted drug delivery, with their ability to selectively deliver therapeutics to cells expressing specific receptors. This enhances intracellular drug delivery, minimizes drug resistance, and reduces toxicity. In this research, we comprehensively evaluate the ADMET (absorption, distribution, metabolism, excretion, and toxicity) properties of various AMPs and CPPs to gain insights into their potential as anticancer agents. The peptide synthesis involved a solid-phase synthesis using a Liberty Microwave Peptide Synthesizer. The peptide purity was confirmed via LC-MS and HPLC methods. For the ADMET screening, computational tools were employed, assessing parameters like absorption, distribution, metabolism, excretion, and toxicity. The cell lines A549 and UM-UC-5 were cultured and treated with 5-FU, CAMPs, and CPPs. The cell viability was measured using the MTT assay. The physicochemical properties analysis revealed favorable drug-likeness attributes. The peptides exhibited potential inhibitory activity against CYP3A4. The ADMET predictions indicated variable absorption and distribution characteristics. Furthermore, we assessed the effectiveness of these peptides alone and in combination with 5-FU, a widely used antineoplastic agent, in two distinct cancer cell lines, UM-UC-5 and A549. Our findings indicate that CAMPs can significantly reduce the cell viability in A549 cells, while CPPs exhibit promising results in UM-UC-5 cells. Understanding these multifaceted effects could open new avenues for antiviral and anticancer research. Further, experimental validation is necessary to confirm the mechanism of action of these peptides, especially in combination with 5-FU.

## 1. Introduction

Traditional anticancer therapies have unfavorable pharmacokinetic properties and physicochemical/biopharmaceutical barriers that limit therapeutic efficacy at the site of action [1,2]. The lack of tumor specificity and ineffective accumulation of drugs in tumors leads to the use of higher doses of therapeutic agents, resulting in high toxicity to healthy normal cells and severe side effects [3,4] A strategy for overcoming these challenges involves the development of new formulations capable of efficient cell penetration. The targeted delivery of drugs to the site of action is essential to overcome the current drawbacks of cancer therapy [3,5]. Peptides have been used as carriers in drug delivery systems to target drug molecules to specific cell types [6,7]. Due to its receptor selectivity, the peptide–drug conjugate is directed to target cells with a higher expression of a specific receptor that recognizes the carrier peptide. Consequently, the drug is delivered to the diseased target cells without affecting the normal cells that lack the targeted receptors [8,9]. This improvement in the intracellular drug delivery and tumor-targeted release reduces drug resistance and toxicity and increases the therapeutic efficacy at the targeted sites of action [10].

Lately, anticancer peptides (ACPs) have emerged as a potential therapeutic strategy for cancer treatment due to their high level of cell penetration, selectivity, and few side effects [11]. Research has been conducted on two types of ACPs: antimicrobial peptides (AMPs) and cell-penetrating peptides (CPPs). AMPs are synthetic or natural peptides with short amino acid sequences (10–60 amino acids) with antimicrobial or anticancer activity [12,13]. Most AMPs are cationic and amphipathic molecules produced by prokaryotic and eukaryotic organisms as part of the host’s innate immune system [14,15]. Indeed, cationic AMPs (CAMPs) disrupt the membranes of microbial cells by interacting with negatively charged phospholipids, leading to the formation of pores in the microbial membrane, which causes the leakage of cytoplasmic components and cell death [13,16]. Moreover, AMPs have been reported to selectively target human tumor cells through their ability to bind the phospholipid phosphatidylserines (PS), which are localized in the outer leaflet of the plasma membrane of cancer cells, leading to necrosis or apoptosis [17].

CPPs are positively charged peptides consisting of five to thirty synthetic or natural amino acids capable of internalizing into different cell types by direct penetration or endocytosis [7,9,12]. They can be classified into three main classes, according to their physicochemical properties: cationic, amphipathic, and hydrophobic. Cationic CPPs are peptides with a high net positive charge associated with the presence of lysine and arginine residues. This leads to the electrostatic interaction between the cationic amino acids and the proteoglycans/phospholipids on the cell membrane, resulting in the cellular penetration of the CPPs. Furthermore, amphipathic CPPs contain amino acids with both polar and non-polar properties that covalently link the hydrophobic domain to a nuclear localization signal that targets peptide cargoes to the cell nucleus through the nuclear pore complex. Finally, hydrophobic CPPs are low net charge peptides containing non-polar amino acids. Due to their high affinity for the hydrophobic domains of cellular membranes, these peptides can translocate across membranes [7,18]. Moreover, due to their low toxicity and rapid cell internalization property, CPPs have been studied as vectors to transport therapeutic drugs across the physiological barriers of cells [9,19]

Another common and promising strategy in cancer treatment involves the strategic combination of drugs. This approach capitalizes on the synergy between traditional chemotherapeutic agents and other compounds, thereby providing a multi-pronged attack on cancer cells. The overarching goal is to significantly enhance the effectiveness of chemotherapy, while simultaneously mitigating its associated side effects and the potential development of drug resistance [20,21,22]. Previous research from our group has identified the synergistic interactions between various peptides, CPPs, and established chemotherapeutic agents such as paclitaxel (PTX), 5-fluorouracil (5-FU), and clotrimazole (CLZ) by enhancing the delivery of chemotherapeutic agents to different cancer cells [23]. In addition, a different study explored the potent anticancer properties of CPP2-thiazole conjugates. These conjugates were investigated in combination with paclitaxel and 5-FU in prostate and colon cancer cells, respectively. This research discovered that CPP2’s N-terminal modification significantly enhanced its anticancer activity. The response to these combinations was not uniform across different cell lines, with PC-3 cells proving to be more responsive to the synergy between CPP2-thiazole conjugates and clotrimazole [24]. This variation underscores the importance of considering cell line-specific responses in developing combination therapies and highlights the potential of these compounds as adjuvants in cancer therapy. Both studies emphasize the intricacy of combination therapy in the context of cancer treatment. They showcase the varying responses of different cancer cell lines to synergistic drug interactions and highlight the role of computational methods in predicting the outcomes of these combinations [25]. One of the advantages of this combination therapy is the potential to use lower doses of chemotherapies, which can help reduce the severe side effects often associated with them. Additionally, this approach may help counteract drug resistance, a significant challenge in cancer treatment [26]. Furthermore, these combinatory approaches offer the opportunity to target specific pathways or mechanisms in cancer cells more effectively. Different peptides may affect various aspects of tumor biology, including growth, migration, or resistance to apoptosis.

**Figure 1:**
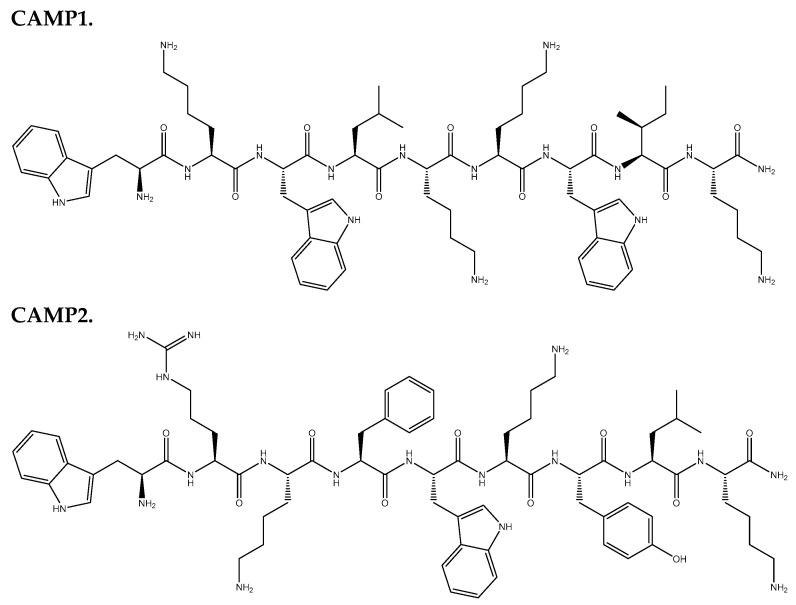

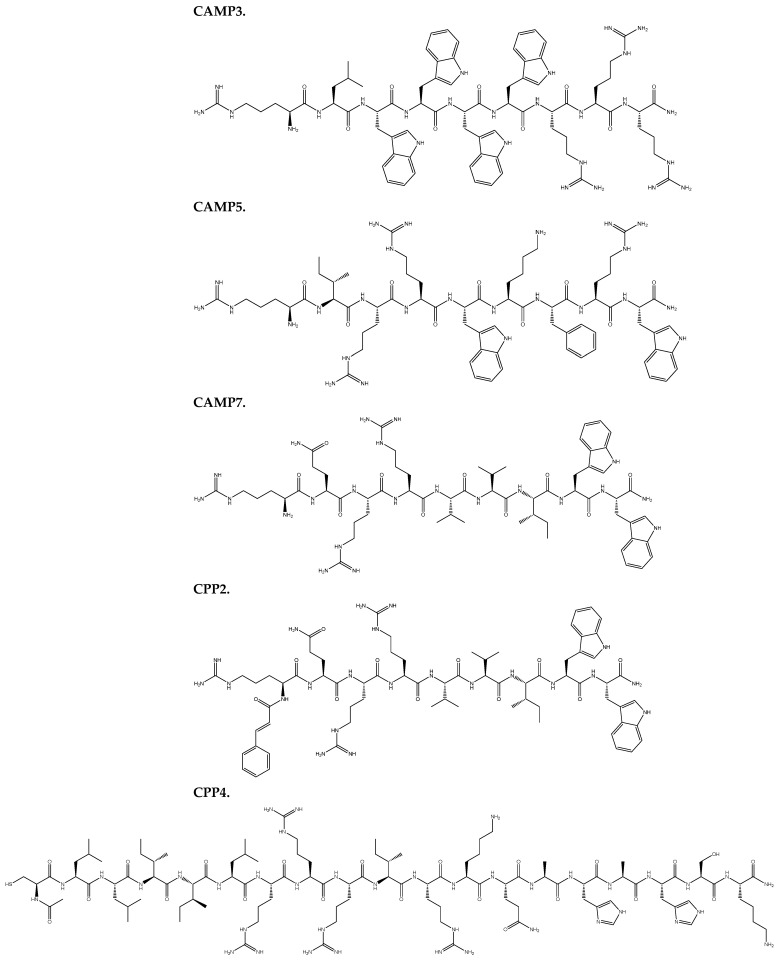
Chemical structures of CAMPs and CPPs used in this project.

In this research, our primary objective was to comprehensively assess the ADMET properties of a range of compounds and discern their potential as anticancer agents based on their specific mechanisms of action. Notably, both CPPs and CAMPs exhibited favorable attributes, positioning them as promising candidates for drug development. Moreover, CAMP1 and CAMP2 were identified as inhibitors of CYP3A4, a critical enzyme in drug metabolism. This discovery can influence drug metabolism and enhance the effectiveness of combination therapies. Subsequently, our investigation delved into assessing various AMPs and CPPs in their capacity to enhance the antineoplastic properties of 5-FU within UM-UC-5 and A549 cancer cell lines. The chemical structures of the peptides used in this study are illustrated in Figure 1. These evaluations encompassed individual peptide assessments and their combination with 5-FU to gauge overall effectiveness.

## 2. Materials and Methods

### 2.1. Peptide Synthesis

Peptide CPP4 was synthesized with a C-terminal amide using solid-phase peptide synthesis (SPPS) and assisted with microwave (MW) energy. A Liberty Microwave Peptide Synthesizer (CEM Corporation, Mathews, NC, USA) was used, following the Fmoc/tBu orthogonal protection scheme. To begin, the resin was pre-conditioned in *N*,*N*-dimethylformamide (DMF) for 15 min and then transferred into the MW-reaction vessel. The initial Fmoc deprotection step involved using 20% piperidine in DMF with 0.1 M 1-hydroxybenzotriazole (HOBt) in two MW pulses: 30 s at 24 W followed by 3 min at 28 W. In both cases, the temperature did not exceed 75 °C. The C-terminal amino acid was then coupled to the deprotected Rink amide resin. This involved using 5 molar equivalents (eq.) of the Fmoc-protected amino acid in DMF (0.2 M), 5 eq. of 0.5 M HBTU/HOBt in DMF, and 10 eq. of 2 M *N*-ethyl-*N*,*N*-diisopropylamine (DIPEA) in *N*-methylpyrrolidone (NMP). The coupling step was conducted for 5 min at 35 W irradiation, with the maximum temperature reaching 75 °C. The remaining amino acids were sequentially coupled in the C → N direction using similar deprotection and coupling cycles. Double-coupling was utilized when coupling lysines incorporated as Fmoc-Lys(Boc)-OH.

N-terminal acetylation was carried out with a mixture of 20 eq. of acetic anhydride and 20 eq. of DIPEA in 2 mL of DMF, for 1 h. If applicable, fatty acid coupling to the N-terminal amino acid was performed manually using 5 eq. of the acid, 5 eq. of PyBOP, and 10 eq. of DIPEA in 2 mL of DMF, for 2 h. Upon completion of the sequence assembly, the peptide was released from the resin, simultaneously removing side-chain protecting groups. This was achieved through a 2-h acidolysis at room temperature using a trifluoroacetic acid (TFA)-based cocktail containing triisopropysilane (TIS) and water as scavengers (in a 95:2.5:2.5 *v*/*v*/*v* ratio).

An LCQ-DecaXP LC-MS system from ThermoFinnigan, equipped with both a DAD detector and an electrospray ionization-ion trap mass spectrometer (ESI/IT MS) was used to carry out the peptide analysis. The Peptide analysis using the HPLC method was performed with a Hitachi-Merck LaChrom Elite system equipped with an RP-18E column (5 μm), a quaternary pump, a thermostatted automated sampler and a diode-array detector (DAD). When necessary, peptides were purified through a preparative HPLC system (LaPrep Sigma, MA, USA), with an LP1100 Quaternary LPG pump injection with a fractionation valve. Final peptides were lyophilized, and their purity was checked with an HPLC system. Peptides with a high purity (>97%) were used.

CAMP peptide amino acid sequences were derived from 9-amino acid peptides and synthesized through standard manual Fmoc solid-phase peptide synthesis (SPPS) [27,28]. This process initiates with the coupling of an N-alfa protected amino acid onto a solid support (resin). In this case, the chosen resin was the Rink Amide MBHA resin LL (100–200 mesh), a polystyrene-based polymer functionalized with 4-methylbenzhydrylamine (MBHA) groups, and further modified with an N-Fmoc-protected (R,S)-2-{4-[amino(2,4 dimethoxyphenyl)methyl]phenoxy} acetic acid linker (Rink-amide linker). The resin’s swelling was achieved by adding *N*,*N-*dimethylformamide (DMF) with continuous stirring. After 20 min, DMF was removed, followed by adding dichloromethane (DCM) for 15 min. Prior to peptide synthesis, the initial removal of the Fmoc group was carried out using 20% piperidine in DMF (3 mL, 1 × 1 min + 1 × 20 min). Following deprotection, the resin was rinsed with DMF (3 mL, 3 × 1 min) and DCM (3 mL, 3 × 1 min), and a Kaiser test was performed. The remaining α-amino acids in the sequence were sequentially linked together through the formation of amide bonds (peptide bonds). The elongation step involved coupling an amino acid to the peptidyl-resin. Before being transferred to the syringe vessel, the amino acids were activated for 5 min using a solution of Fmoc-AA-OH (5 eq.), coupling agent O-(benzotriazol-1-yl)-*N*,*N*,*N*′,*N*′-tetramethyluronium hexafluorophosphate (HBTU, 5 eq.), and base DIPEA (10 eq.) in DMF. The activated amino acid solution was then transferred to the syringe to react with the previously deprotected resin or peptidyl-resin for 1 h with continuous stirring. Once the coupling was complete, the peptidyl-resin was washed with DMF (3 mL, 3 × 1 min) and DCM (3 mL, 3 × 1 min), and a Kaiser test was performed. When the test result was negative (yellow), the deprotection step was applied using a solution consisting of 20% piperidine in DMF (3 mL, 1 × 1 min + 1 × 20 min). After this, the resin was washed with DMF (3 mL, 3 × 1 min) and DCM (3 mL, 3 × 1 min), and another Kaiser test was conducted. If confirmed positive (dark blue), the next Fmoc-AA-OH was coupled using the previously described method. This cycle of coupling N-Fmoc amino acids and deprotection steps was repeated until the desired peptide sequence was achieved. The resulting CAMP(n) peptides were solubilized in 10% aqueous acetic acid and purified through RP-MPLC (Reversed-Phase Medium-Pressure Liquid Chromatography), using a gradient of ACN in water with 0.05% TFA as the eluent (15% to 35%). The collected fractions were then analyzed using the HPLC method to identify those containing the conjugate with a purity exceeding 97%. These fractions were subsequently pooled, lyophilized, and stored at −22 °C until needed.

The Peptide CPP2 sequence was also produced following the standard Fmoc-SPPS strategy. The Fmoc deprotection was performed using 20% piperidine in the presence of 0.1 M HOBt during 3 × 3 min. The acylation was carried out using 5 eq. of amino acids dissolved in DMF together with 4.8 eq. HCTU, 10 eq. DIPEA, and 5 eq. HOBt for 2 × 15 min. The removal of the protecting groups was performed using a cocktail mixture containing 95% TFA, 2.5% H_2_O, 2.5% TIS for 3 h. Retention times were acquired by an HPLC-DAD analytical system, with a C18 column, using ACN in water with 0.1% TFA as eluent, in gradient mode (10–80%), for 18 min, at a flow rate of 1 mL/min and detection at λ = 220 nm. The peptide showed a degree of purity of 98%.

### 2.2. ADMET Screening of the Cell-Penetrating Peptides and the Cationic Antimicrobial Peptides

Two computational methods were used to thoroughly analyze the physicochemical parameters of the cell-penetrating peptides and the cationic antimicrobial peptides to gather knowledge about the absorption, distribution, metabolism, excretion, and toxicity (ADMET) parameters of the peptide sequences. The .mol files of the structures were imported to the ADMET predictor software (version 10.4, Simulations Plus, Inc., Lancaster, CA, USA) and the Simplified Molecular Input Line Entry System (SMILES) structural format of the peptides was generated to use in ADMETlab2.0 (https://admetmesh.scbdd.com/, accessed on 4 September 2023), using Convert, a molecule file format converter (via ChemAxon JChem) [29]). The ADMET predictor tool was employed to assess various ADMET parameters. The comprehensive analysis included human intestinal absorption (HIA), mutagenicity, carcinogenicity, central nervous system penetration, drug-induced liver injury (DILI), cytochrome p450 enzyme inhibition, clearance, half-life, and skin sensitization, among others. ADMETlab 2.0 was utilized for these predictions. To further refine the assessment, within this software, the presence of Pan-Assay Interference Compounds (PAINS) and undesirable reactive compounds were analyzed using the PAINS rules and Pfizer rules. This process assisted in determining the compounds that might exhibit stronger anticancer activity and the associated mechanisms of action. Both tools also provided predictions of pharmacokinetic parameters. We used user manuals and literature guides to guide the analysis of the calculations made with this tool [30,31,32]. This knowledge improved our comprehension of the pharmacokinetic characteristics of the peptides.

### 2.3. Cell Lines and Cell Culture Conditions

A549 adenocarcinomic human alveolar basal epithelial cells, UM-UC-5 urothelial bladder cancer cell lines, and MRC-5 human normal lung fibroblast cells (all from ATCC, American Type Culture Collection, Manassas, VA, USA) were cultured in Dulbecco’s Modified Eagle Medium (DMEM) medium (Gibco^®^, Grand Island, NY, USA), supplemented with 10% fetal bovine serum (FBS, Gibco^®^, Grand Island, NY, USA) and 1% (*v*/*v*) penicillin/streptomycin (Sigma-Aldrich^®,^ Steinheim, Germany), maintained at 37 °C and 95% humidified air in a 5% CO_2_ environment. Cells were cultured in monolayers in T25 cm^2^ flasks (ThermoScientific^®^, Waltham, MA, USA) and cell media were replaced every 2–3 days. At 80% confluency, cells were subcultured. For the subculture process, 0.25% trypsin-ethylenediaminetetraacetic acid (EDTA, Sigma-Aldrich^®^, Steinheim, Germany), was added.

All described procedures were performed under aseptic conditions in a flow chamber.

### 2.4. In Vitro Drug Protocol

The potential of the antineoplastic drug 5-FU, five cationic antimicrobial peptides (CAMPs), and two cell-penetrating peptides (CPPs) was analyzed in UM-UC-5 and A549 cell lines. Cells were treated with drugs in concentrations ranging from 0.1 to 100 μM (for 5-FU) and 0.1 to 50 μM (for CAMPs and CPPs) for 48 h, and cell survival was evaluated using the MTT assay. Based on these results, a dose–response curve was obtained, and the half maximal inhibitory concentration (IC_50_) value was calculated. IC_50_ values > 100 μM were not considered.

### 2.5. Cell Viability Assay

The cell viability was measured using the MTT (Thiazolyl blue tetrazolium bromide; cat. no. M5655; Sigma-Aldrich; Merck KGaA, Darmstadt, Germany) assay in 96-well plates. For the protocol, 5 × 10^3^ cells in 200 µL of medium were grown per well for 24 h at 37 °C. Then, cells were treated with CAMPs and CPPs in the following concentrations: 0.1, 1.0, 5.0, 10, 25, and 50 µM. The cell viability of the combination with increasing concentrations of CAMPs and CPPs with the IC_50_ of 5-Fluorouracil (5-FU, 5 µM for A549 and 12.5 µM for UM-UC-5) was later analyzed. The controls were treated with 0.1% H_2_O and 0.1% DMSO (vehicles). After incubation for 48 h at 37 °C, cell media were aspirated and 100 µL of MTT solution (0.5 mg/mL in PBS) was added to each well. After incubation for 3 h at 37 °C protected from light, MTT solution was removed from each well and 100 µL of DMSO was added to each well to solubilize the formazan crystals. Absorbance was measured on a Tecan Infinite M200 plate reader (Tecan Group Ltd., Männedorf, Switzerland) at 570 nm. The cell viability was calculated relative to the absorbance of carrier controls. Average values were obtained by performing three independent cell viability assays (n = 3).

## 3. Results

### 3.1. Physicochemical Properties and Medicinal Chemistry

A useful method for evaluating molecules in biochemical assays is ADMET screening, which enables us to strategically choose experiments and focus our efforts on the most promising compounds. Based on the assessment of these physicochemical properties and the prescribed rules and thresholds, it is evident that the compounds exhibit multiple characteristics well-aligned with drug-likeness criteria. These favorable attributes include appropriate values for molecular weight, hydrogen bond acceptors and donors, rotatable bonds, ring count, heteroatoms, TPSA, solubility, logP, and logD (Table 1). It is worth noting that there is a variation in the LogP values between different calculation tools, with the CPP2 logP closely matching the prediction made by the ADMET predictor.

Moreover, the compounds demonstrate several advantageous properties in light of the provided information and the specified criteria. These include a notably high Fsp3 value, compliance with the Pfizer rule, and a notable absence of alerts for Pan-Assay Interference Compounds (PAINS), and chelation-related concerns. The Lipinski rules, commonly known as the Rule of Five (Ro5), serve as guidelines to identify drug-like compounds based on specific molecular properties. The Ro5 suggests that a compound is more likely to be orally bioavailable if it adheres to the following criteria: the molecular weight (MW) is less than 500 Da; the calculated octanol–water partition coefficient is (cLogP) less than five; the number of hydrogen bond donors (HBDs) is less than five; and the number of hydrogen bond acceptors (HBAs) is less than ten. However, it is essential to note that the Ro5 is not a strict rule but rather a heuristic tool to filter out compounds unlikely to be absorbed in the gastrointestinal (GI) tract. Exceptions to the Ro5, known as beyond the Rule of Five (bRo5) compounds, challenge traditional boundaries. The bRo5 space is expansive and diverse, encompassing compounds with the following properties: the MW is greater than 500 Da; the cLogP is less than 0 or greater than 7.5; the number of HBDs is greater than five; the number of HBAs is greater than ten; the polar surface area (PSA) greater than 200 Å^2^; and the number of rotatable bonds (NRotB) is greater than twenty.

This chemical space holds promise for drug discovery, providing access to novel targets, including protein–protein interactions (PPIs), intracellular targets, and unique binding sites. Additionally, it presents opportunities to design compounds with enhanced selectivity, potency, and pharmacokinetics. Despite its potential benefits, the bRo5 space introduces challenges like poor solubility, permeability, stability, and toxicity. Strategies to navigate these challenges include the following: (1) utilizing natural products or derivatives, which often possess favorable biological activity and bioavailability despite being bRo5 compounds; (2) designing macrocycles or cyclic structures to reduce conformational entropy and enhance binding affinity; (3) conjugating bRo5 compounds with metal catalysts, peptides, or other moieties to enable targeted drug activation or release, reducing systemic exposure and toxicity; (4) employing advanced formulation and delivery technologies to improve solubility, permeability, and stability. Naylon et al. further emphasize the importance of understanding structure–property relationships, especially in the context of cyclic peptides targeting protein–protein interactions. This highlights the challenges of achieving the oral bioavailability of cyclic peptides while violating the Ro5, showcasing examples like cyclosporin A as bRo5 compounds. The authors suggest various strategies for designing and synthesizing bRo5 cyclic peptides, underlining this chemical space’s vast potential and challenges [33].

The Pfizer rule is a set of guidelines for assessing drug-likeness and optimizing pharmaceutical compounds. The GSK rule is a set of guidelines followed by GlaxoSmithKline in drug discovery and development. The Golden Triangle is a concept in medicinal chemistry that highlights the balance between molecular size, lipophilicity, and polarity. The Pan-Assay Interference Compounds is a list of compounds known to interfere with many bioassays and often produce false-positive results. At last, the ALARM NMR Rule is a rule used to identify potentially problematic compounds in nuclear magnetic resonance (NMR) spectroscopy studies. It is worth noting that some compounds warrant attention, as they do not fully meet the criteria for the QED and SAscore. Further, they do not adhere to the GSK and Golden Triangle rules and may incur violations of the Lipinski rules.

The ADMETlab web server was also used to predict the following: (1) absorption—CaCO_3_ permeability (CaCO_2_), P-gp inhibitor/substrate (P-gp), human intestinal absorption (HIA), 20% bioavailability (F20%), and 30% bioavailability (F30%)); (2) distribution—plasma protein binding (PPB), blood–brain barrier (BBB), and volume distribution (VD); (3) metabolism—CYP inhibition and substrate; (4) excretion—half-life (T_1/2_) and clearance (CI) and; (5) toxicity—the hERG potassium channel inhibition (cardiotoxicity), H-HT (human hepatotoxicity), AMES (Ames mutagenicity) and SkinSen (skin sensitization), among others. The properties of CaCO_2_, VD, PPB, CI, and T_1/2_ were expressed numerically, whereas the rest of the pharmacokinetic parameters were expressed categorically.

Table 2 presents the results obtained in the ADMETlab web server. The findings that provide evidence of the peptides’ potential inhibitory activity in tumor cell lines are particularly significant for this study. The Caco-2 permeability results provide insights into how these peptides may be absorbed in the body. Peptides with “good permeability” (such as CPP2, CAMP3, CAMP5, and CAMP7) are more likely to be efficiently absorbed, which can be important for their potential inhibitory activity in tumor cell lines. Peptides with “low permeability” (such as CPP4, CAMP1, and CAMP2) may face challenges in terms of absorption, and this factor should be considered when assessing their potential effects on tumor cells.

All the peptides are highly likely to be substrates of P-glycoprotein (Pgp) and have a high potential to be transported by Pgp. Regarding the Pgp inhibitor capacity, none of the peptides are predicted to be inhibitors. They are not expected to interfere with the activity of Pgp, a membrane protein involved in drug transport. However, CAMP1 and CAMP2 fall into the category “-”, which is not trustable. In the ADMET predictor, we obtained the same results. This information is essential when considering how these peptides might interact with Pgp and their potential inhibitory activity in tumor cell lines.

The human intestinal absorption (HIA) parameter is considered an essential prerequisite for the compound’s apparent efficacy. The results indicate poor absorption. The human oral bioavailability results of 20% and 30% (F20% and F30%) indicate the efficiency of the drug delivery to the systemic circulation, which are also poor.

Regarding distribution, almost all compounds have excellent PPB, VD, and Fu values, suggesting favorable distribution characteristics. Only CPP4 presents a low VD. However, the value obtained for the VD of CPP4 is very different from the value obtained in the calculations of the ADMET predictor, where we even obtained a positive value, which is similar to the other peptides.

Drug metabolism reactions can be broadly divided into two groups based on the chemical properties involved in biotransformation: phase I, which includes oxidative reactions, and phase II, which comprises conjugative reactions. Among these, the human cytochrome P450 family, a subset of phase I enzymes, comprises 57 isozymes. Remarkably, these isozymes play a pivotal role in metabolizing approximately two-thirds of known drugs in humans, with a significant 80% attributed to just five isozymes: 1A2, 3A4, 2C9, 2C19, and 2D6. Only CAMP1 and CAMP2 have been indicated as inhibitors of CYP3A4. In the ADMET predictor, all the CPPs and CAMPs are considered inhibitors of at least three CYPs. For all compounds, we obtained poor clearance and medium to poor T_1/2_ values in the ADMETlab2.0 properties analysis.

The toxicity profiles presented in Table 2 were predicted using ADMETlab2.0. Some profiles need to be highlighted. Among these profiles, certain findings warrant special attention. Specifically, concerning human hepatotoxicity (H-HT), the results for CPP2 indicate a medium level of activity (++), implying that some molecules within this compound group possess the potential for hepatotoxicity. However, it is advisable not to trust predictions for all the other compounds. Respiratory toxicity is the main safety issue. Calculations suggest a high probability of respiratory toxicity for CAMP1, CAMP2, and CAMP3.

The nuclear receptor estrogen receptor ligand-binding domain (NR-ER-LBD) term refers to a specific functional domain within the estrogen receptor, a type of nuclear receptor involved in mediating the effects of the hormone estrogen. The ligand-binding domain (LBD) is a crucial region within the nuclear receptor that interacts with estrogen and other ligands, initiating signaling pathways and gene expression changes in response to hormonal cues. CPP2, CPP4, and CAMP7 will likely interfere with the estrogen receptor function. The interaction of compounds with the NR-ER-LBD is closely linked to cancer, particularly hormone-responsive cancers like breast cancer.

The peroxisome proliferator-activated receptor gamma (NR-PPAR-gamma) regulates glucose and lipid metabolism. CPP2 and CAMP1 have a high probability of being active, which suggests a potential effect on glucose and lipid metabolism and may have therapeutic implications. CAMP2 is not as likely but has a good probability of interfering with NR-PPAR-gamma. This suggests a potential effect on glucose and lipid metabolism and may have therapeutic implications.

The assessment of the antioxidant response element (SR-ARE) evaluates the ability of molecules to activate the antioxidant response element, which is crucial in mitigating oxidative stress. The results show a medium activity level (++) for CPP2 and CAMP1, indicating that some molecules can activate this protective pathway against oxidative stress. CPP4 has a medium probability (--) of being inactive in activating the antioxidant response element (ARE) signaling pathway. This result suggests that CPP4 may not contribute significantly to the protective response against oxidative stress mediated by this pathway.

CAMP1 is the only compound classified as positive (+++) for both the ATPase family AAA domain-containing protein 5 (SR-ATAD5) and the heat shock factor response element (SR-HSE), indicating a higher probability of being active in these pathways. SR-ATAD5 is associated with DNA damage response, and SR-HSE is associated with stress conditions.

CPP2 and CAMP5 have a moderate probability (++) of activating the signal response-matrix metalloproteinase (SR-MMP). The matrix metalloproteinases (MMPs) are a family of enzymes that play a role in the degradation of the extracellular matrix and are involved in various physiological and pathological processes, including tissue remodeling, wound healing, and cancer metastasis.

At last, we have the probability of activating the tumor suppressor protein 53 (SR-p53). The compounds CPP2, CAMP1, and CAMP2A are classified as SR-p53 positive (++), (+++), and (+++), respectively. This indicates a higher probability of activating p53 for these compounds. The activation of p53 is associated with DNA damage response and cell fate regulation.

Table 3 provides an overview of the ADMET predictor analysis, encompassing mechanisms not covered by the ADMETlab2.0 web server. Within all the ADMET predictor results, two key findings deserve special attention.

Firstly, these compounds have exhibited promising antiviral capabilities. HIVI-TC [log(mol/L)] reveals the pIC50 value, signifying the potency in inhibiting HIV-1 integrase 3′-processing, while HIVI-ST [log(mol/L)] represents the pIC50 value for suppressing the HIV-1 integrase strand transfer. However, we must highlight that the values underlined in these results were flagged in red by the ADMET predictor. In the ADMET prediction, red values typically signal that a compound’s predicted properties or characteristics may fall into a range considered less favorable or potentially problematic based on established criteria. Consequently, it is imperative to underscore that these findings are predictive and require experimental validation to ascertain the actual inhibitory activity of the compounds. Furthermore, it is crucial to note that, except CAMP1, all compounds are substrates of the breast cancer resistance protein (BCRP), with only CAMP1 classified as an inhibitor of the BCRP. This could represent another avenue for investigating the anticancer activity, warranting further exploration.

**Figure 2:**
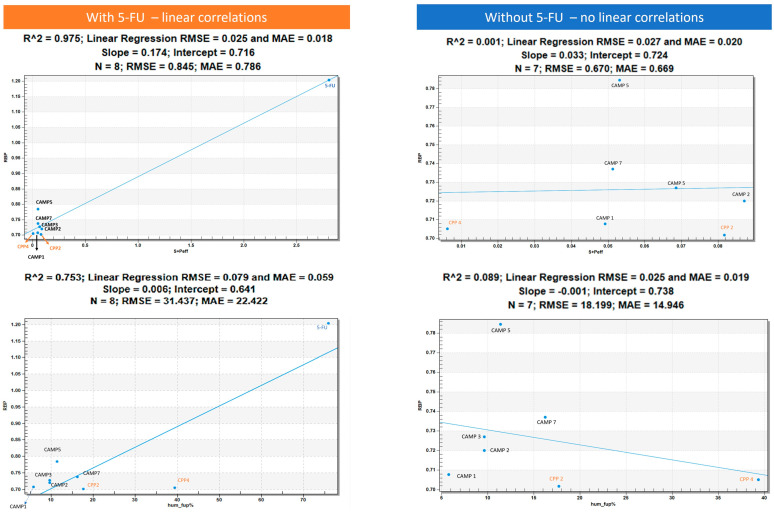
On the left we can see the linear positive relation between the ratio of blood plasma (RBP) and human effective jejunal permeability (S + Peff) and between RBP and human fraction unbound in plasma percentage (hum_fup%). On the right side, we removed the 5-FU; the R-squared (R^2^) value drops to 0.001 and 0.089, indicating that there is essentially no linear relationship between the remaining compounds.

In Figure 2, we illustrate a positive linear correlation between the ratio of blood plasma (RBP) and human effective jejunal permeability (S + Peff), as well as between the RBP and the percentage of human fraction unbound in plasma (hum_fup%). The graphs, which incorporate 5-FU, exhibit high R-squared (R^2^) values, signifying a robust association between these variables. Furthermore, the low values of the root mean square error (RMSE) and mean absolute error (MAE) indicate precise predictions, underscoring the strength and accuracy of this linear relationship. Notably, the 5-FU drug stands out with notably higher values for both parameters. On the other hand, CAMP and CPP, situated in the bottom left, are closely grouped, highlighting their similar relationship. This valuable insight sheds light on how changes in S + Peff and hum_fup% can impact the RBP for these drugs, offering critical implications for drug development and potential clinical applications. However, when we exclude 5-FU from the analysis, the R^2^ values plummet to 0.001 and 0.089. These diminished values indicate an almost non-existent linear connection among the remaining compounds. It becomes apparent that variations in S + Peff do not effectively account for or predict changes in the RBP for these four drugs. In essence, the linear regression model proves to be inadequate for describing the relationship between S + Peff and the RBP for these particular compounds.

### 3.2. Cell Viability Assays

The effectiveness of a drug in inhibiting cell growth can significantly vary between different tumoral cell lines, and this knowledge plays a crucial role in tailoring treatments to specific cancer types. In this context, Table 4 displays the IC50 values for the compounds tested in two distinct tumoral cell lines, UM-UC-5 and A549, across a 48-h experimental duration. The IC50 value represents the drug concentration necessary to inhibit cell growth by 50%. In the UM-UC-5 cell line, 5-FU demonstrates a potent IC50 value of approximately 4.21 μM, showcasing its effectiveness in restraining cell proliferation. In contrast, A549 cells exhibit a slightly lower IC50 value of 2.42 μM for 5-FU, indicating its effectiveness in this specific cell line.

CAMP1, when assessed in the UM-UC-5 cell line, exhibits a significantly high IC50 value, surpassing 100 μM, suggesting its inability to efficiently inhibit cell growth in this context. Conversely, the IC50 value in the A549 cell line is 12.39 μM, signifying a moderate level of effectiveness in restraining cell proliferation. For CAMP2, IC50 values diverge between the two cell lines. UM-UC-5 cells demonstrate an IC50 value of 21.61 μM, while A549 cells exhibit a notably lower IC50 value of 5.77 μM. This indicates that CAMP2 is notably more effective at inhibiting cell growth in A549 cells. CAMP3 displays IC50 values exceeding 100 μM in the UM-UC-5 cell line, denoting limited efficacy in inhibiting cell growth. In the A549 cell line, the IC50 value is 17.63 μM, suggesting a moderate potency in restraining cell proliferation.

Similarly, CAMP5 showcases IC50 values exceeding 100 μM in UM-UC-5 cells, signifying a low effectiveness. In A549 cells, the IC50 value is 19.65 μM, indicating a moderately inhibitory potential. CAMP7, when tested in both UM-UC-5 and A549 cell lines, reveals IC50 values exceeding 100 μM, implying limited efficacy in curtailing cell growth in both scenarios.

For CPP2, the UM-UC-5 cell line demonstrates a relatively high potency with an IC50 value of 5.47 μM, effectively inhibiting cell growth. Conversely, the A549 cell line exhibits a reduced effectiveness with an IC50 value exceeding 100 μM. In both UM-UC-5 and A549 cell lines, CPP4 displays IC50 values exceeding 100 μM, suggesting its ineffectiveness in inhibiting cell growth.

Collectively, these IC50 values provide valuable insights into the varying effectiveness of these compounds in restraining cell proliferation across distinct cell lines. While certain compounds like 5-FU and CPP2 exhibit potency in specific cell lines, others, such as CAMP1 and CAMP7, display limited effectiveness in both cell types. These findings are fundamental for comprehending the potential applicability of these drugs in precise cancer treatments.

**Figure 3:**
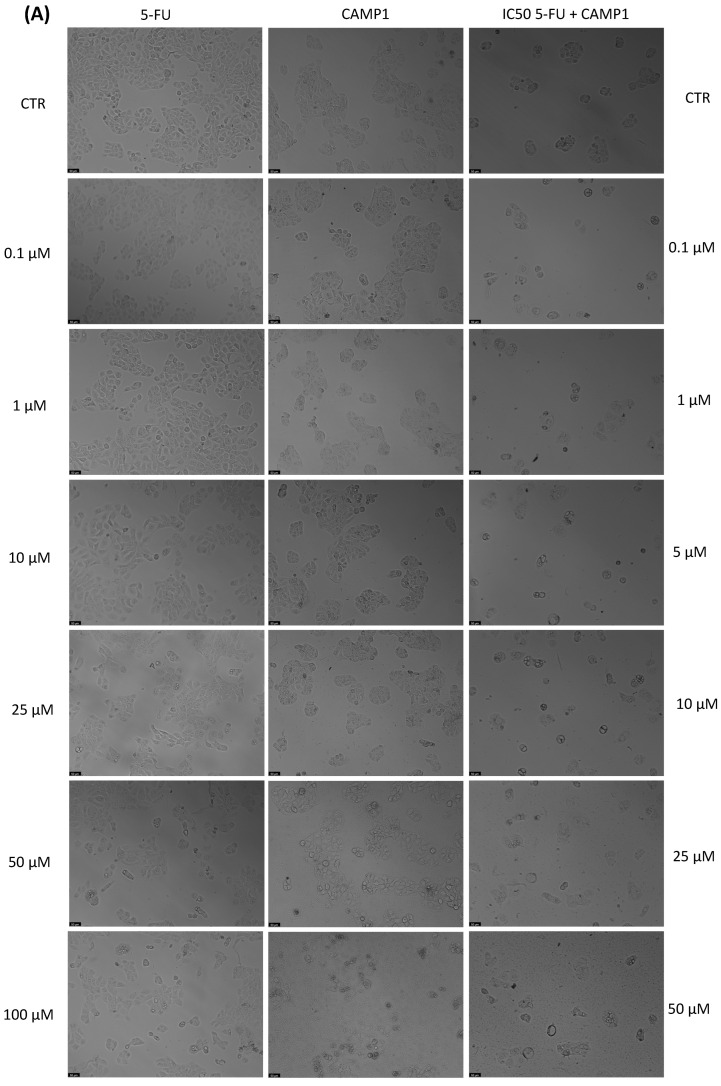

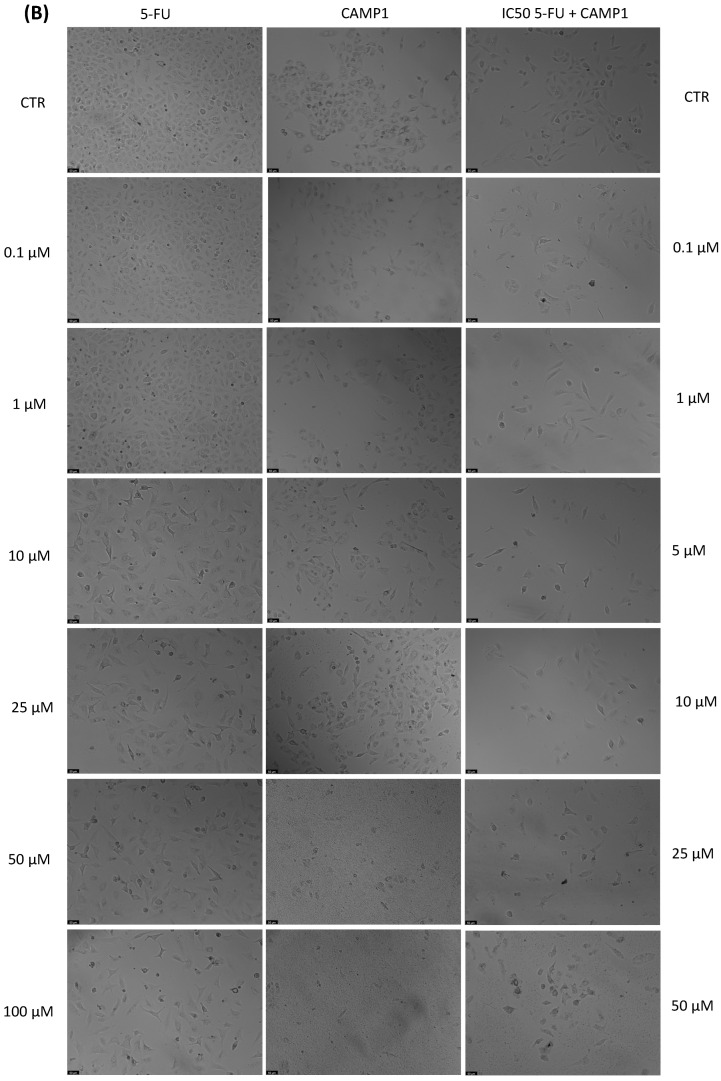
Morphological analysis of 5-FU and CAMP1 alone and in combination in (**A**) UM-UC-5 and (**B**) A549 cells. Scale bar: 50 µm.

**Figure 4:**
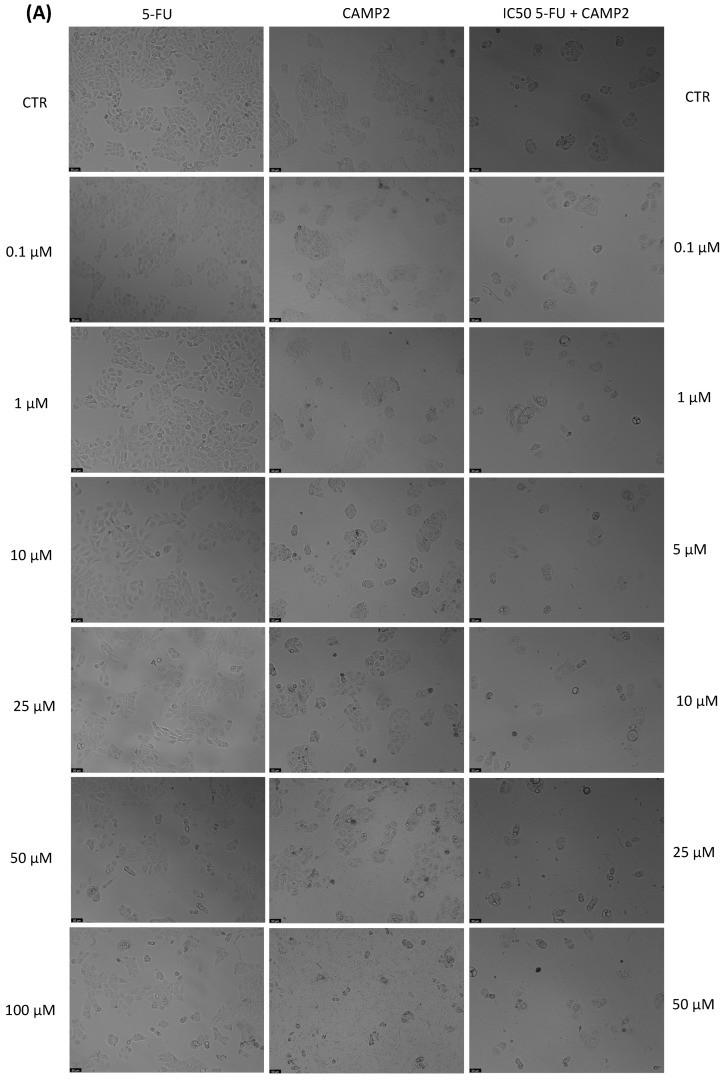

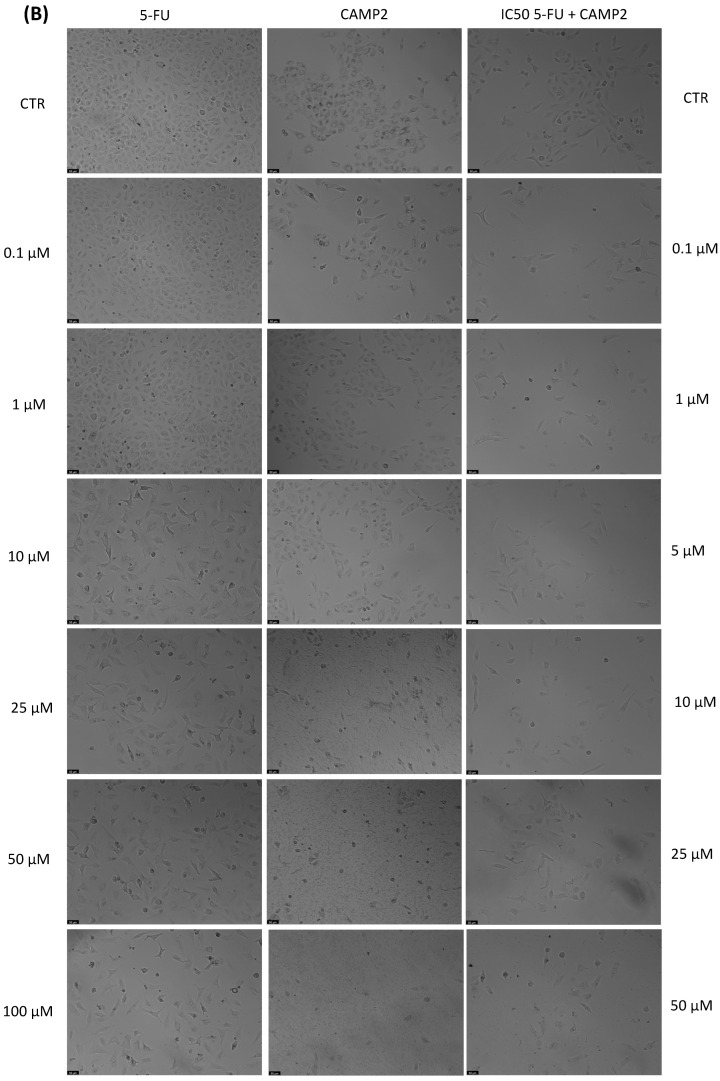
Morphological analysis of 5-FU and CAMP2 alone and in combination in (**A**) UM-UC-5 and (**B**) A549 cells. Scale bar: 50 µm.

**Figure 5:**
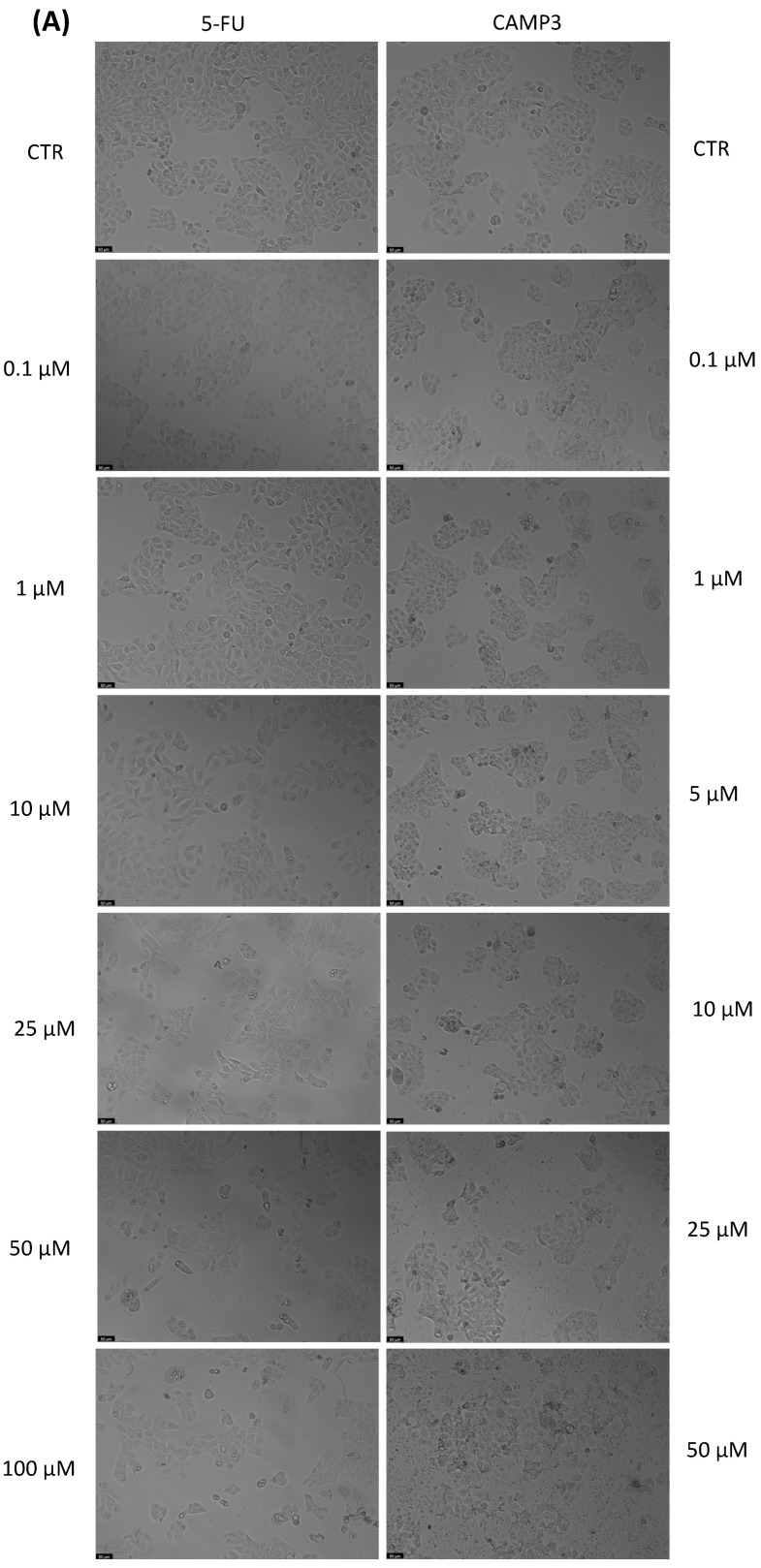

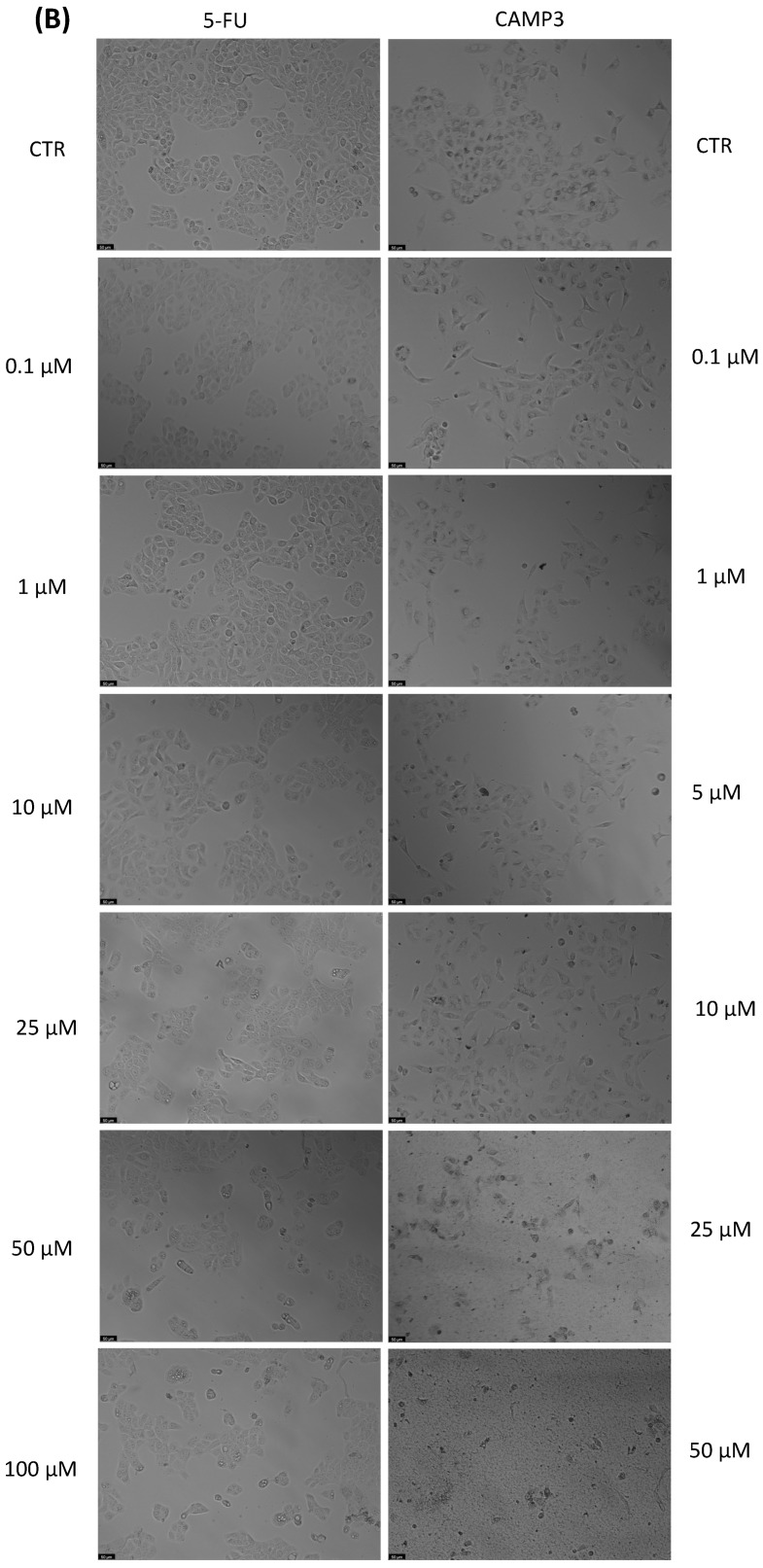
Morphological analysis of 5-FU and CAMP3 alone in (**A**) UM-UC-5 and (**B**) A549 cells. Scale bar: 50 µm.

**Figure 6:**
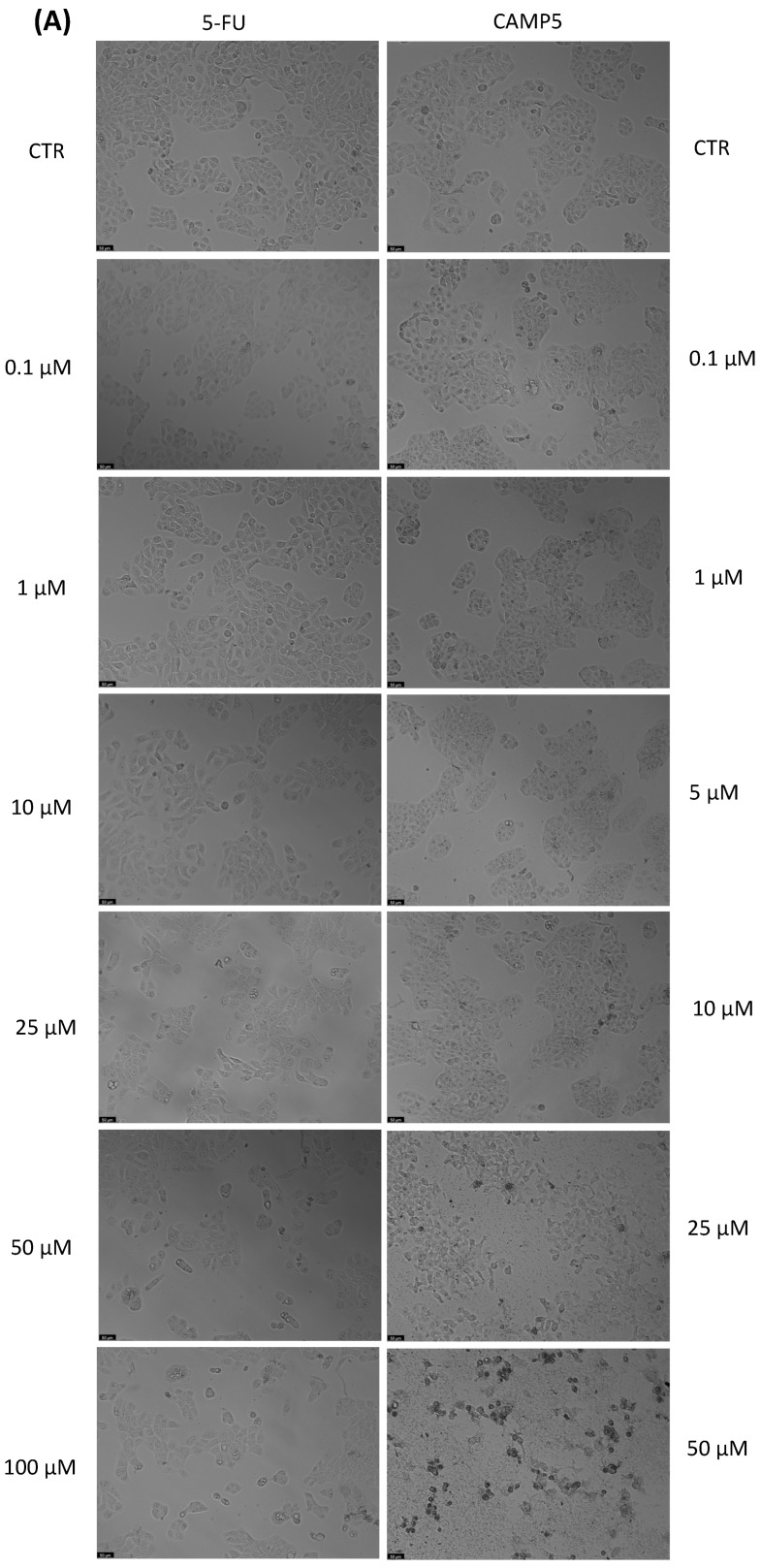

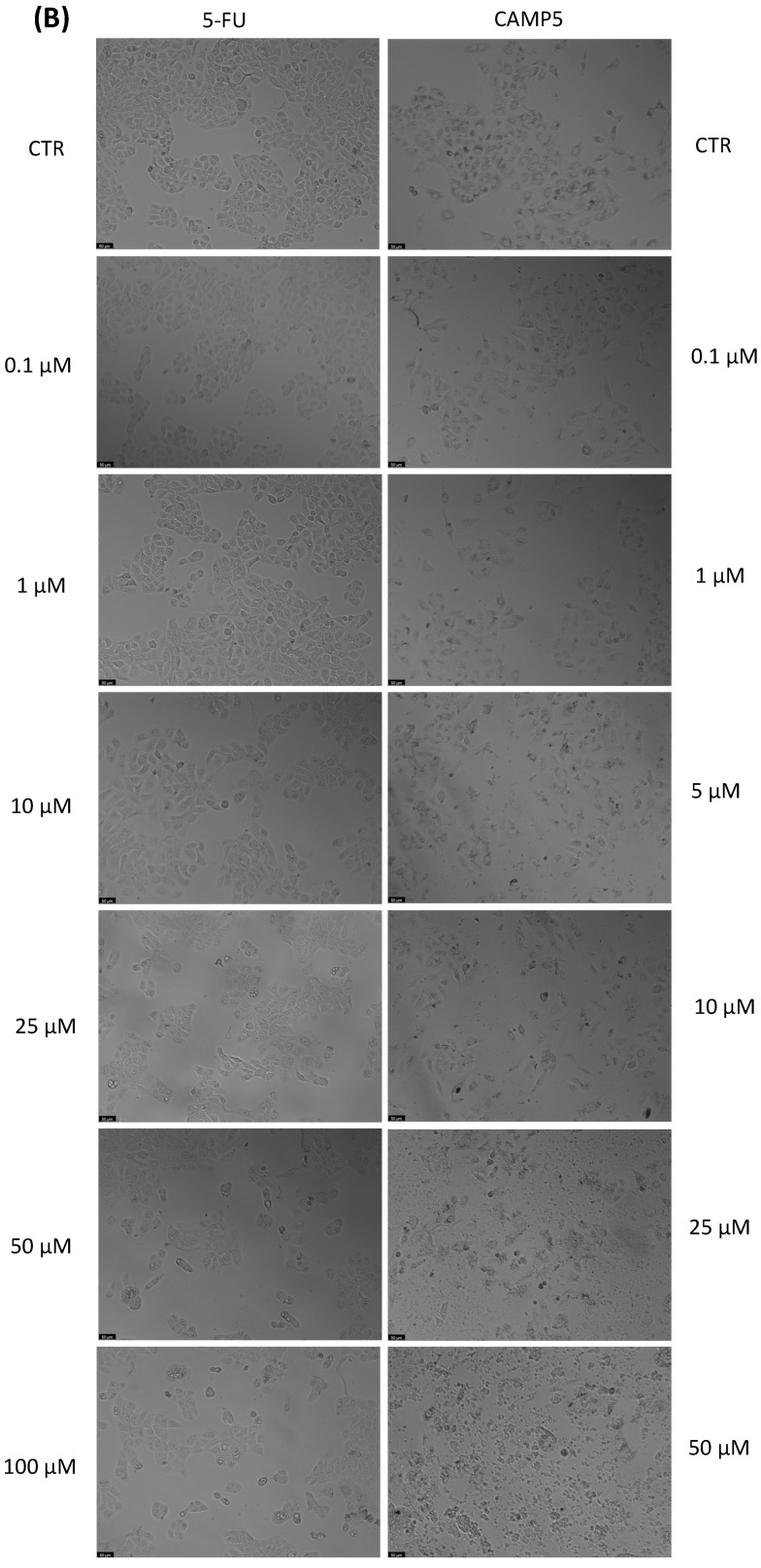
Morphological analysis of 5-FU and CAMP5 alone in (**A**) UM-UC-5 and (**B**) A549 cells. Scale bar: 50 µm.

**Figure 7:**
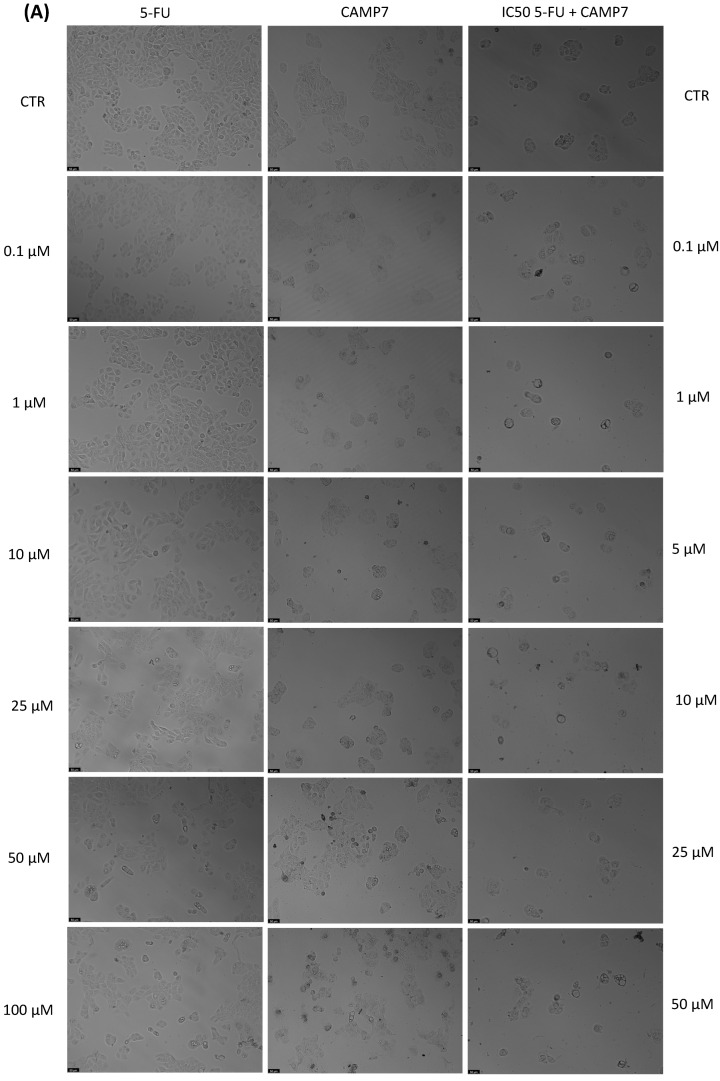

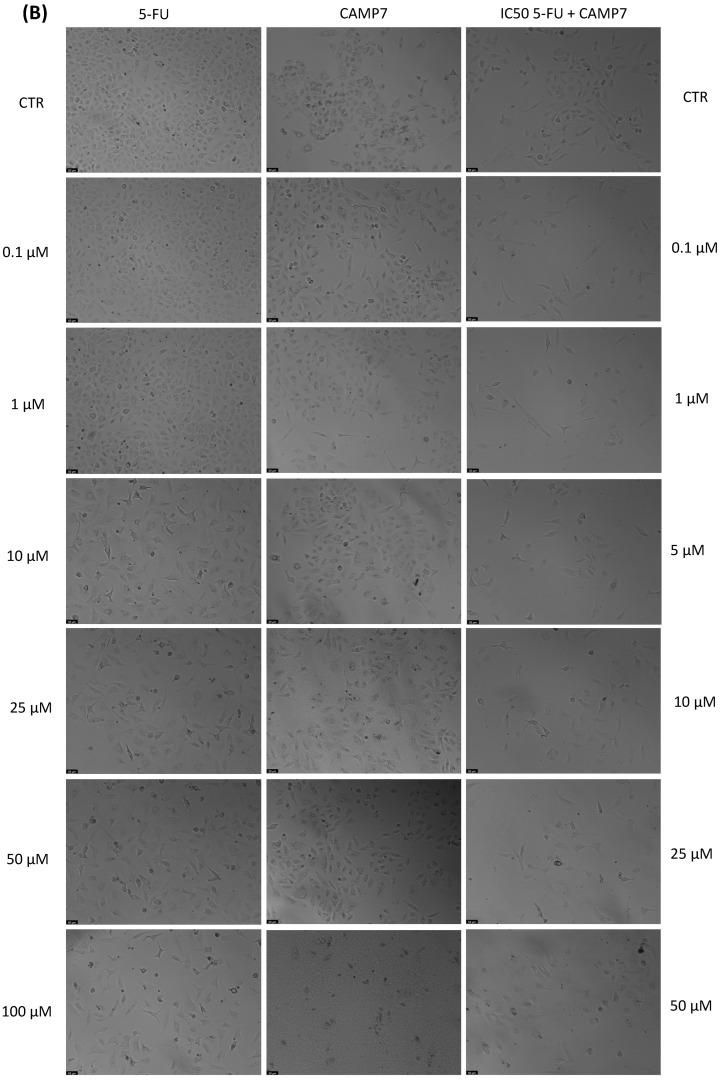
Morphological analysis of 5-FU and CAMP7 alone and in combination in (**A**) UM-UC-5 and (**B**) A549 cells. Scale bar: 50 µm.

**Figure 8:**
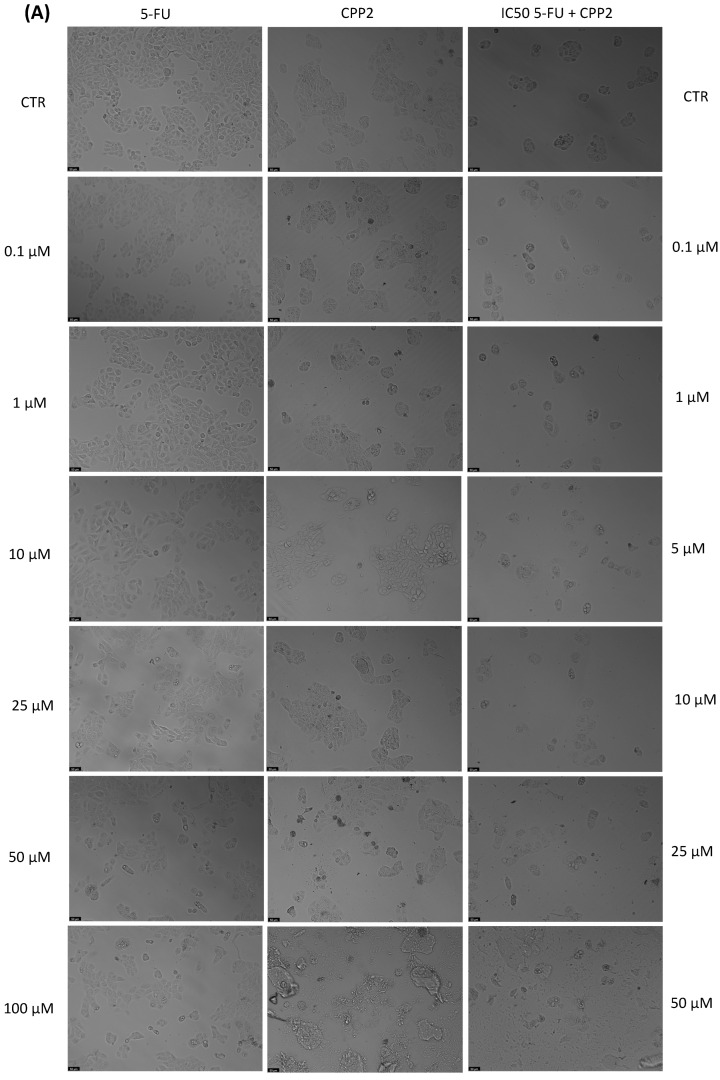

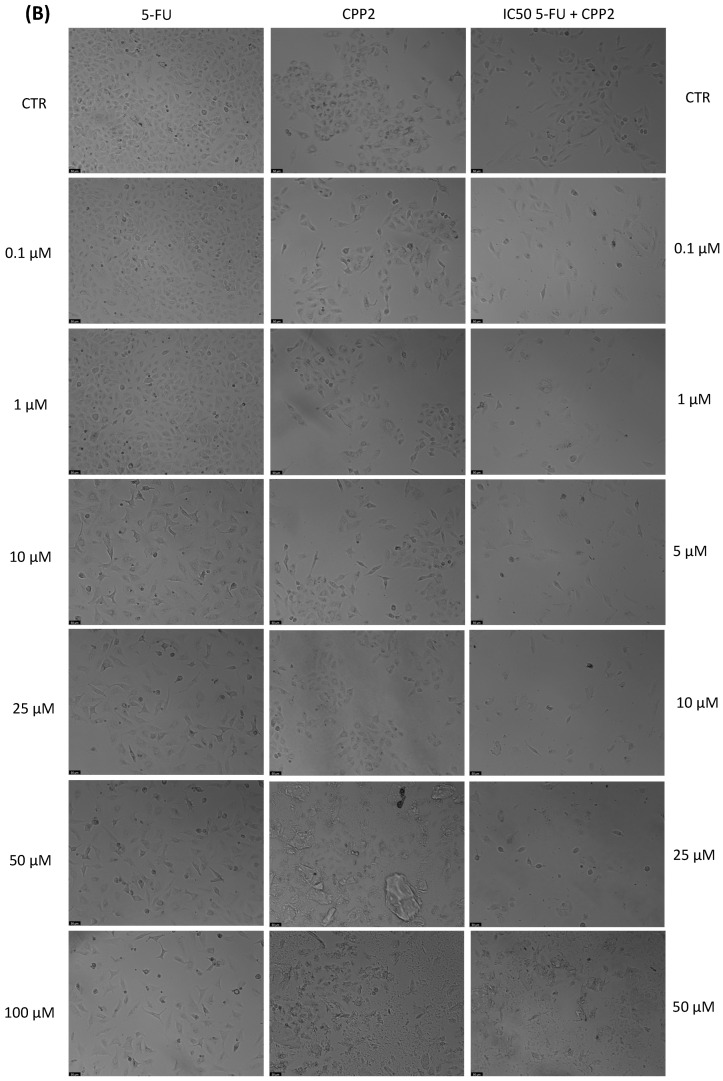
Morphological analysis of 5-FU and CPP2 alone and in combination in (**A**) UM-UC-5 and (**B**) A549 cells. Scale bar: 50 µm.

**Figure 9:**
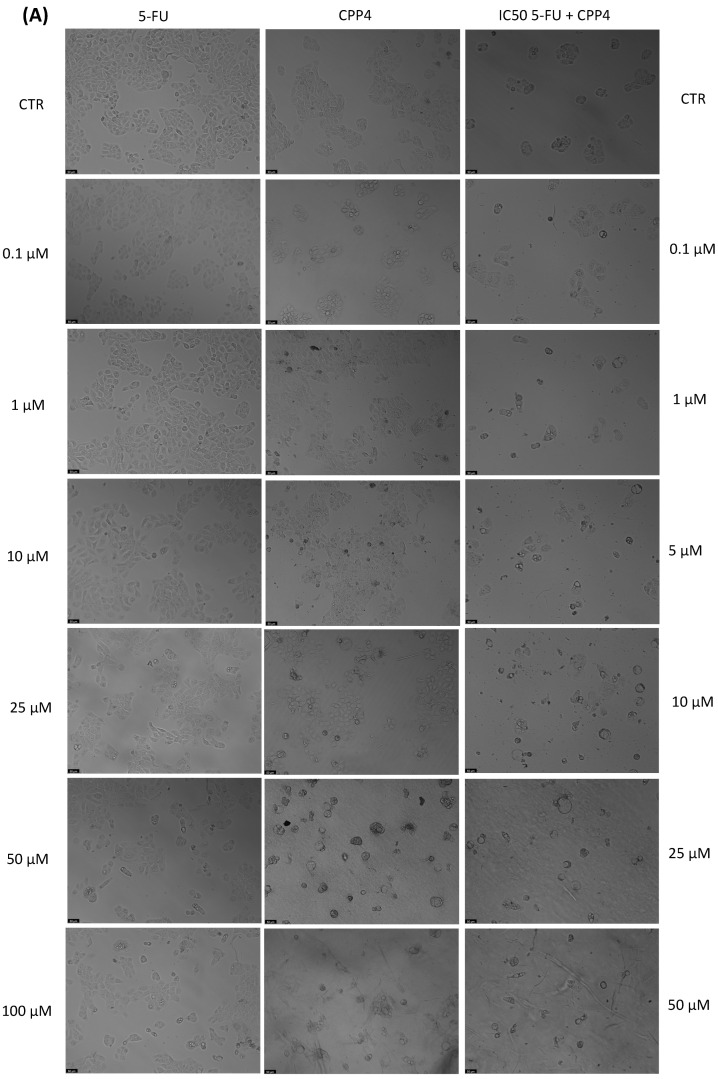

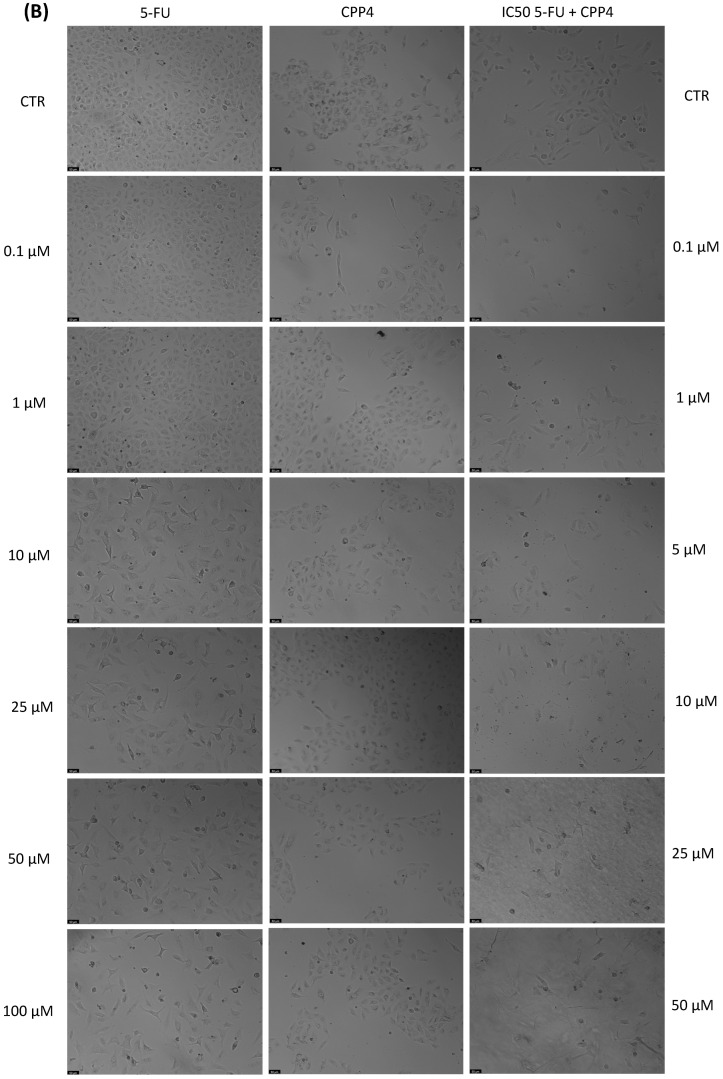
Morphological analysis of 5-FU and CPP4 alone and in combination in (**A**) UM-UC-5 and (**B**) A549 cells. Scale bar: 50 µm.

With the aim of better understanding the effectiveness of the peptides alone or combined with a reference drug for cancer treatments, and using two different cell lines, we proceeded with microscopic observations for the visual representation of cellular morphology and behavior. This allows us to observe how cancer cells respond to different compounds at a microscopic level. Phenotypic screening, based on these visual observations, can identify potential drug candidates that might not have been predicted through other methods. This is because cancer is a highly heterogeneous disease, meaning that different types of cancer cells can coexist within the same tumor. Cell images help us to understand this diversity, enabling us to identify drugs that can target various subpopulations of cancer cells. Also, with these observations, we can obtain insights into how drugs affect cellular processes. This information is crucial for understanding the mechanisms of action of known and potential drugs. It can also reveal unexpected interactions, leading to the discovery of new applications for existing drugs. The findings detailed in Figure 3, Figure 4, Figure 5, Figure 6, Figure 7, Figure 8 and Figure 9 are complemented by supplementary images (refer to Figure S1 for the morphological evaluation of MRC-5 cells treated with Peptides CAMP1, CAMP2, CAMP3, CAMP5, CAMP7, CPP2, and CPP4). These supplementary figures provide additional insights into the observed observations.

## 4. Discussion

This analysis has been conducted to assess the ADMET properties of each compound and gain insights into their potential anticancer efficacy based on the activated mechanisms. Table 1 outlines the physicochemical properties of the compounds and their alignment with drug-likeness criteria. While the compounds exhibit favorable attributes regarding molecular weight, hydrogen bond acceptors and donors, rotatable bonds, and more, some aspects, such as the QED, SAscore, and adherence to certain rules, may need further attention. Overall, these properties suggest that the compounds have potential as drug candidates.

Table 2 presents a range of pharmacokinetic properties, including absorption, distribution, metabolism, excretion, and toxicity. Notably, the Caco-2 permeability results indicate that some peptides (e.g., CPP2, CAMP3, CAMP5, and CAMP7) are efficiently absorbed, which is essential for their potential inhibitory activity in tumor cell lines. However, others (e.g., CPP4, CAMP1, and CAMP2) may face absorption challenges. Additionally, all peptides are likely substrates of P-glycoprotein (Pgp), which may impact their transport in the body. Pgp is a membrane protein involved in drug transport. When peptides are substrates of Pgp, it means that Pgp can potentially transport these peptides in and out of cells. This transport capability can affect how effectively these peptides reach and interact with cancer cells. In anticancer therapy, the ability of drugs or compounds to reach cancer cells and exert their effects is crucial. If Pgp is highly active in transporting a compound out of cancer cells, it can reduce the intracellular concentration of the compound, potentially limiting its anticancer efficacy. On the other hand, if Pgp transports the compound into cancer cells, it may enhance its intracellular concentration and therapeutic potential. So, considering whether a peptide is a substrate of Pgp is a pharmacokinetic consideration that can influence the bioavailability and distribution of these peptides in the body, which, in turn, can impact their effectiveness as anticancer agents. However, none of the peptides used are predicted Pgp inhibitors. Moreover, the HIA results suggest poor absorption properties and that oral bioavailability is low. Most compounds exhibit favorable distribution characteristics, although CPP4 has a low volume of distribution. Drug metabolism predictions indicate poor clearance and low half-life values for all compounds. These properties may help ensure that the compounds primarily reach tumor tissues, are distributed effectively within tumors, and remain in circulation long enough to exert their anticancer effects.

CAMP1 and CAMP2 have been indicated as inhibitors of CYP3A4, a key enzyme in drug metabolism. Inhibiting this enzyme could affect the metabolism of various drugs, potentially enhancing the efficacy of combination therapies with these peptides. This inhibition may also reduce the clearance of certain drugs, leading to higher drug concentrations in the body, which can benefit cancer treatment. Regarding H-HT, while CPP2 shows a medium level of activity for hepatotoxicity (++), it is crucial to be cautious about its potential liver toxicity. Understanding the hepatotoxicity risk is essential, especially if these peptides are administered systemically. Further investigation is needed to assess their safety profiles.

In the context of respiratory toxicity, it is noteworthy that CAMP1, CAMP2, and CAMP3 exhibit a high probability, suggesting potential lung-related side effects. However, it is important to clarify that our assessment primarily centers around the potential anticancer mechanisms of these peptides. Notably, our findings indicate more favorable results for the A549 cell line, particularly with CAMP1 and CAMP2, while showing fewer promising outcomes for these CAMPs in the UM-UC-5 cell line. These differential responses underscore the importance of considering cell line-specific effects when evaluating the potential therapeutic utility of these peptides for anticancer activity.

For the NR-ER-LBD parameter, CPP2, CPP4, and CAMP7 are predicted to interfere with the estrogen receptor (ER) function. This is particularly relevant in hormone-responsive cancers like breast cancer, where ER plays a crucial role. These peptides might affect ER-mediated signaling pathways, potentially inhibiting tumor growth in ER-positive cancers. To delve deeper into their effects, it is essential to consider specific cell line responses. Notably, CPP2 and CPP4 have demonstrated promising anticancer activity in the UM-UC-5 cell lines while showing no activity in the A549 cell lines. Although there are conflicting results, it has been demonstrated that estrogen receptors play a role in urothelial carcinogenesis, cancer progression, and regulating chemosensitivity in bladder cancer [34,35]. Nonetheless, ERs also play an important role in non-small cell lung cancer (NSCLC) [36,37]. The varying outcomes across cell lines underscore the complexity of these interactions and the importance of exploring them further to harness their therapeutic potential effectively.

As mentioned earlier, CPP2 and CAMP1 are likely to be active in interacting with PPAR-gamma. This activation may impact glucose and lipid metabolism, potentially leading to therapeutic implications in cancers associated with dysregulated metabolism. They also exhibit a medium activity level in activating the antioxidant response element. This indicates their potential to mitigate oxidative stress, a factor associated with cancer progression and resistance to therapies. These peptides might enhance the body’s ability to combat oxidative stress and support the effectiveness of cancer treatments. Concerning the SR-ATAD5 and SR-HSE, CAMP1 stands out as positive (+++) for both of these pathways, indicating a higher probability of activity. These pathways are associated with DNA damage response and stress conditions, suggesting that CAMP1 might enhance the cellular response to DNA damage, a critical aspect of cancer therapy.

CPP2 and CAMP5 have a moderate probability (++) of activity in the SR-MMP pathway. MMPs play a role in tissue remodeling and cancer metastasis. These peptides might impact the extracellular matrix, potentially affecting cancer cell invasion and metastasis. CPP2, CAMP1, and CAMP2A are classified as SR-p53 positive (++ to +++). The activation of p53 is associated with DNA damage response and cell fate regulation. These peptides might enhance the tumor-suppressing activity of p53, which can lead to cancer cell death.

These peptides exhibit diverse mechanisms of action that could contribute to their potential as anticancer agents. However, it is crucial to emphasize that these predictions are based on computational models and require rigorous experimental validation in preclinical and clinical studies to confirm their actual efficacy and safety in treating cancer.

Additionally, the compounds display promising antiviral capabilities, as indicated by their pIC50 values for inhibiting HIV-1 integrase 3′-processing and strand transfer. It is worth noting that while some of these values are flagged in red by the ADMET predictor, suggesting potential concerns, we conducted a literature review to provide context. The pIC50 value, as a measure of inhibitor potency, is calculated as the negative logarithm of the IC50 value, representing the concentration at which the inhibitor reduces enzyme activity by 50%. In our research, we found that the minimum and maximum reported pIC50 values for inhibiting HIV-1 integrase 3′-processing are as follows: minimum—the lowest pIC50 value reported is 4.06, corresponding to an IC50 value of 63 μg/mL for the extract of Bauhinia holophylla; maximum—the highest pIC50 value reported is 6.06, corresponding to an IC50 value of 8.8 μg/mL for the ethanolic extract (BH-HT) of Bauhinia holophylla [38]. It is important to acknowledge that these values are based on a limited number of studies.

In the context of cancer research, it is interesting to explore how some peptides, like certain CPPs and CAMPs, can inhibit HIV integrase. For example, a hexapeptide derived from the N-terminal domain of HIV-1 integrase has demonstrated the inhibition of integrase activity at low micromolar concentrations, affecting both cytoplasmic and nuclear events mediated by integrase [39]. Moreover, peptide-functionalized gold nanoparticles were designed to target the active site of integrase and block its strand transfer activity [40].

However, it is important to note that not all CPPs and CAMPs have inhibitory effects on integrase; some may even enhance its activity or facilitate cellular delivery. Interestingly, research has suggested a possible link between integrase inhibitors and cancer incidence or progression in individuals living with HIV. For instance, a cohort study found no association between cancer risk and cumulative integrase inhibitor exposure [41]. Laboratory studies have shown that integrase inhibitors can modulate gene expression in cancer development and metastasis in HIV-infected cells [42]. Hence, a hypothesis for further studies of the mechanisms of action of CPPs and CAMPs could be the capacity of inhibiting HIV integrase. While our focus has primarily been on antiviral properties, the potential implications for cancer research warrant further investigation. It is crucial to consider both antiviral and anticancer mechanisms when exploring the multifaceted effects of these compounds.

At last, all compounds except CAMP1 are substrates of the breast cancer resistance protein (BCRP), with CAMP1 being an inhibitor of the BCRP. This is interesting, especially when considering its potential use in tumor cell lines, alone or in combination with other compounds. The BCRP is known for its role in drug resistance, particularly in cancer cells. When the BCRP is highly active, it can pump out various drugs from cancer cells, reducing the effectiveness of chemotherapy or other therapeutic agents. By inhibiting the BCRP, CAMP1 may enhance the intracellular concentration of other drugs used in cancer treatment. This could lead to increased drug efficacy and improved outcomes in tumor cell lines. CAMP1’s inhibitory effect on the BCRP opens up possibilities for combination therapy. It could be used alongside traditional chemotherapy drugs or targeted therapies to improve their intracellular retention and effectiveness. This combination approach may help to overcome drug resistance mechanisms which often emerge during cancer treatment. The specific mechanisms by which CAMP1 interacts with the BCRP and its effects on different tumor types should be thoroughly investigated to determine the full scope of its potential as an adjuvant in cancer treatment.

In the context of an in vitro analysis assessing the effectiveness of peptides in cancer cells, the observations derived from cell images can play a pivotal role in the repurposing of drugs for cancer treatment. We can identify potential peptide candidates, understand drug mechanisms of action, and ultimately accelerate the development of effective treatments for cancer patients. In this project, compounds of varying effectiveness for inhibiting cell growth across different tumoral cell lines were identified, emphasizing the importance of tailoring treatments to specific cancer types.

The microscopic images (Figure 3, Figure 4, Figure 5, Figure 6, Figure 7, Figure 8 and Figure 9) are important for understanding that there is a significant correlation between what is observed and the IC50 values in Table 4, which presents IC50 values for the tested compounds in UM-UC-5 and A549 cell lines over a 48-h experimental period. In UM-UC-5 cells, 5-FU exhibits a potent IC50 value of approximately 4.21 μM, indicating its effectiveness in restraining cell proliferation. Conversely, A549 cells show a slightly lower IC50 value of 2.42 μM for 5-FU, highlighting its efficacy in this specific cell line. CAMP1 displays a high IC50 value (>100 μM) in UM-UC-5 cells, suggesting limited effectiveness in inhibiting cell growth. In contrast, in A549 cells, the IC50 value is 12.39 μM, signifying a moderate level of effectiveness. For CAMP2, UM-UC-5 cells show an IC50 value of 21.61 μM, while A549 cells exhibit a notably lower IC50 value of 5.77 μM, indicating greater effectiveness in inhibiting cell growth in A549 cells. CAMP3 demonstrates IC50 values exceeding 100 μM in UM-UC-5 cells, denoting limited efficacy. In A549 cells, the IC50 value is 17.63 μM, indicating moderate potency. Similarly, CAMP5 exhibits IC50 values exceeding 100 μM in UM-UC-5 cells, signifying low effectiveness. In A549 cells, the IC50 value is 19.65 μM, indicating a moderately inhibitory potential. CAMP7, tested in both UM-UC-5 and A549 cell lines, shows IC50 values exceeding 100 μM, implying limited efficacy in inhibiting cell growth.

CPP2 demonstrates a relatively high potency in UM-UC-5 cells with an IC50 value of 5.47 μM, effectively inhibiting cell growth. Conversely, A549 cells show reduced effectiveness with an IC50 value exceeding 100 μM. In both UM-UC-5 and A549 cell lines, CPP4 displays IC50 values exceeding 100 μM, indicating ineffectiveness in inhibiting cell growth. Overall, these IC50 values offer valuable insights into the varying effectiveness of these compounds in restraining cell proliferation across distinct cell lines. While some compounds like 5-FU and CPP2 show potency in specific cell lines, others like CAMP1 and CAMP7 demonstrate limited effectiveness in both cell types. These findings are crucial for understanding the potential applicability of these drugs in precise cancer treatments.

It is clear that the peptides under investigation possess multifaceted properties and mechanisms that may contribute to their potential as anticancer agents. However, these potentialities need further validation. It is crucial to acknowledge several limitations that should be considered when interpreting the results and planning further research. The ADMET properties and the anticancer efficacy predictions are primarily based on computational models. These models have inherent limitations and may not fully represent the complex biological processes and interactions in a living organism. Further experimental validation is essential to confirm the accuracy of these predictions.

The use of 5-fluorouracil (5-FU) in cancer treatment has been enhanced through various delivery systems and methodologies, including the use of macromolecules, nanocomposites, and gold complexes [43]. Therefore, the cooperation of 5-FU with AMPs and CPPs could be a promising strategy for cancer treatment. Some studies have shown that conjugating 5-FU with AMPs or CPPs can improve its anticancer activity and reduce toxicity. For example, the combination of 5-FU with selenium nanoparticles has shown enhanced anticancer activity, with great selectivity between cancer and normal cells [44]. Furthermore, the functionalization of cetuximab onto a hybrid nanoplatform has been found to effectively encapsulate and selectively deliver 5-FU against colorectal cancer cells, while also inhibiting cell migration and invasion [45]. The use of cell-penetrating peptides as delivery systems for cancer therapeutics, including 5-FU, has also been explored, with a focus on increasing drug uptake in tumor cells [46].

## 5. Conclusions

In conclusion, our research has unveiled the multifaceted potential of various peptides as anticancer agents, combining in silico and in vitro assessments. While our computational insights are promising, extensive experimental research is needed to validate the true mechanisms of action of these compounds and harness their full therapeutic potential. Our preliminary results revealed that CAMPs exhibited a superior capacity to reduce cell viability in A549 cells, whereas CPPs showcased promising results in UM-UC-5 cells. We found that CAMP1 and CAMP2 suppress CYP3A4, an important enzyme involved in drug metabolism, which may improve combination treatments. Nonetheless, these findings call for further, in-depth analyses to fully elucidate the mechanisms and potentials of these compounds in anticancer therapy. The variability in IC50 values highlights the intricate nature of these compounds’ interactions with different cancer types, emphasizing the importance of tailored treatments. These findings underscore the complexity of developing targeted cancer therapies and the necessity of personalized medicine. Moving forward, further research can delve deeper into the molecular mechanisms underlying the variable effectiveness of these compounds in specific cell lines, advancing the field of oncology and its mission to combat cancer effectively and efficiently.

## Figures and Tables

**Table 1:** Physicochemical and medicinal chemistry properties of CAMPs and CPPs.

Physicochemical Properties	CPP2	CPP4	CAMP1	CAMP2	CAMP3	CAMP5	CAMP7
MW	1426.810	2352.430	1313.810	1352.790	1498.830	1401.840	1296.770
Volume	1446.566	2337.240	1361.332	1390.506	1508.578	1415.361	1303.851
Density	0.986	1.006	0.965	0.973	0.994	0.990	0.995
nHA	33	61	26	28	35	34	32
nHD	27	46	23	25	32	32	28
nRot	54	106	51	51	54	56	50
nRing	5	2	6	6	8	5	4
MaxRing	9	5	9	9	9	9	9
nHet	33	62	26	28	35	34	32
fChar	0	0	0	0	0	0	0
nRig	41	35	39	42	53	39	33
Flexibility	1.317	3.029	1.308	1.214	1.019	1.436	1.515
Stereo Centers	10	22	10	9	9	10	10
TPSA	567.860	1016.310	453.360	496.180	615.170	609.610	564.780
logS	−3.341	−0.969	−2.664	−3.268	−3.907	−3.197	−3.350
logP	1.171	−0.856	3.632	2.263	1.127	−0.826	0.123
logD	0.919	0.127	3.133	1.710	0.606	−0.105	0.085
Medicinal chemistry							
QED	0.010	0.013	0.024	0.014	0.013	0.011	0.012
SAscore	6.393	8.247	5.872	5.954	6.390	6.319	6.061
Fsp3	0.457	0.699	0.522	0.457	0.392	0.478	0.525
MCE-18	96.000	116.000	94.000	94.000	118.000	90.000	86.000
NPscore	0.132	0.085	0.084	0.224	0.155	0.219	0.182
Lipinski Rule	Rejected	Rejected	Rejected	Rejected	Rejected	Rejected	Rejected
Pfizer Rule	Accepted	Accepted	Accepted	Accepted	Accepted	Accepted	Accepted
GSK Rule	Rejected	Rejected	Rejected	Rejected	Rejected	Rejected	Rejected
Golden Triangle	Rejected	Rejected	Rejected	Rejected	Rejected	Rejected	Rejected
PAINS	0 alert(s)	0 alert(s)	0 alert(s)	0 alert(s)	0 alert(s)	0 alert(s)	0 alert(s)
ALARM NMR Rule	1 alert(s)	2 alert(s)	0 alert(s)	1 alert(s)	0 alert(s)	0 alert(s)	0 alert(s)

MW: molecular weight; nHA: number of hydrogen acceptors; nHD: number of hydrogen donors; nRot: number of rotatable bonds; nRing: number of rings; MaxRing: maximum ring size; nHet: number of heteroatoms; fChar: fraction of carbon atoms; nRig: number of rigid bonds; TPSA: topological polar surface area; logS: logarithm of solubility; logP: logarithm of partition coefficient; logD: logarithm of distribution coefficient; QED: quantitative estimate of drug-likeness; SAscore: synthetic accessibility score; Fsp3: fraction of sp3 hybridized carbon atoms; MCE-18: molecular complexity enhancement factor 18; NPscore: natural product-likeness score.

**Table 2:** ADMETlab2.0 properties analysis. Concrete predictive values are provided for those endpoints predicted by the regression models, such as Caco-2 permeability, plasma protein binding, etc. For the endpoints predicted by the classification models, such as Pgp-inhibitor, hERG Blocker, etc., the prediction probability values are transformed into six symbols: 0–0.1 (---), 0.1–0.3 (--), 0.3–0.5 (-), 0.5–0.7 (+), 0.7–0.9 (++), and 0.9–1.0 (+++). The token ‘+++’ or ‘++’ represents the molecule is more likely to be toxic or defective, while ‘---’ or ‘--’ represents nontoxic or appropriate. It is not recommended to trust predictions symbolized by ‘+’ or ‘-’ (probably values in 0.3–0.7), which require further assessment.

Property	CPP2	CPP4	CAMP1	CAMP2	CAMP3	CAMP5	CAMP7
Absorption							
Caco-2 permeability	−4.322 (Good permeability)	−5.992 (Low permeability)	−5.941 (Low permeability)	−5.666 (Low permeability)	−4.367 (Good permeability)	−4.935 (Good permeability)	−4.656 (Good permeability)
Pgp inhibitor	---	---	-	-	---	---	---
Pgp substrate	+++	+++	+++	+++	+++	+++	+++
Human intestinal absorption (HIA)	+++	+++	+++	+++	+++	+++	++
Bioavailability	+++	+++	+++	+++	+++	+++	+++
Distribution							
Plasma protein binding (PPB)	79.405%	38.325%	39.841%	42.057%	78.992%	43.690%	54.342%
Volume of distribution	0.426	−0.049 (low)	0.667	0.611	0.497	0.556	0.484
Blood–brain barrier (BBB)	---	---	---	---	---	---	---
Fraction unbound in plasms (fu)	6.375%	24.403%	38.525%	39.135%	12.596%	31.970%	26.794%
Metabolism							
CYP 1A2/2C19/2C9/2D6/3A4 inhibitor	---	---	CYP3A4	CYP3A4	---	---	---
CYP 1A2/2C19/2C9/2D6/3A4 substrate	---	---	---	---	---	---	---
Excretion							
Clearance (CL)	2.003 (poor)	−0.150 (poor)	2.969 (poor)	1.909 (poor)	2.029 (poor)	1.658 (poor)	1.697 (poor)
Half-life (T_1/2_)	0.375	0.892	0.776	0.495	0.607	0.649	0.498
**Toxicity**							
hERG blockers	---	---	--	--	---	---	---
human hepatotoxicity (H-HT)	++	+	-	+	-	--	+
Drug-induced liver injury (DILI)	---	---	---	---	---	---	---
AMES Toxicity	---	---	--	--	---	---	---
Skin Sensitization	---	---	--	--	---	---	-------
Carcinogenicity	---	---	---	---	---	---	---
Respiratory Toxicity	++	++	+++	+++	+++	++	++
NR-AR	---	---	---	---	---	---	---
NR-AR-LBD	---	---	---	---	---	---	---
Aryl hydrocarbon Receptor (AhR)	---	---	-	---	-	---	---
NR-Aromatase	---	---	+	--	--	---	---
NR-ER	---	---	--	-	--	--	--
NR-ER-LBD	++	++	---	--	+	+	++
NR-PPAR-gamma	+++	--	+++	++	---	---	+
SR-ARE	++	--	++	+	-	+	+
SR-ATAD5	+	--	+++	---	---	---	---
SR-HSE	+	---	+++	-	---	---	---
SR-MMP	++	-	+	+	+	++	+
SR-p53	++	-	+++	++	-	--	--

NR-AR: nuclear receptor androgen receptor; NR-AR-LBD: nuclear receptor androgen receptor ligand-binding domain; aryl hydrocarbon receptor (AhR): a protein involved in regulating the body’s response to environmental toxins and pollutants; NR-aromatase: nuclear receptor aromatase; NR-ER: nuclear receptor estrogen receptor; NR-ER-LBD: nuclear receptor estrogen receptor ligand-binding domain; NR-PPAR-gamma: nuclear receptor peroxisome proliferator-activated receptor gamma; SR-ARE: signal response-antioxidant response element; SR-ATAD5: signal response-ATPase family AAA domain-containing protein 5; SR-HSE: signal response-heat shock factor response element; SR-MMP: signal response-matrix metalloproteinase; SR-p53: signal response-tumor suppressor protein 53.

**Table 3:** ADMET predictor results of the antiviral inhibition and breast cancer resistance protein. The underlined values indicate that the model (AMDET predictor) does not provide a reliable estimate for predictions outside the model’s scope. No confidence estimate is provided for out-of-scope predictions.

Mechanism	CPP2	CPP4	CAMP1	CAMP2	CAMP3	CAMP5	CAMP7
HIVI-ST	4.095	4.536	3.804	4.241	3.967	3.723	3.791
HIVI-TC	4.927	5.363	4.373	4.517	4.421	4.386	4.725
BCRP substrate	Yes	Yes	No (51%)	Yes	Yes	Yes	Yes
BCRP inhibition	No	No	Yes	No (60%)	No	No	No

**Table 4:** IC 50 values of 5-FU, CAMP1, CAMP2, CAMP3, CAMP5, CAMP5, CPP2, and CPP4 in tumoral (A549 and UM-UC-5) cells for 48 h.

Drug/Peptide	Cell Line	IC_50_ (μM)
5-FU	UM-UC-5	4.21
	A549	2.42
CAMP1	UM-UC-5	>100
	A549	12.39
CAMP2	UM-UC-5 A549	21.61 5.77
CAMP3	UM-UC-5 A549	>100 17.63
CAMP5	UM-UC-5 A549	>100 19.65
CAMP7	UM-UC-5 A549	>100 >100
CPP2	UM-UC-A A549	5.47 >100
CPP4	UM-UC-5 A549	>100 >100

## Data Availability

No new data were created or analyzed in this study. Data sharing is not applicable to this article.

## Supplementary Materials

The following supporting information can be downloaded at: https://www.mdpi.com/article/10.3390/jfb14120565/s1, Figure S1: Morphological evaluation of MRC-5 cells treated with Peptides CAMP1, CAMP2, CAMP3, CAMP5, CAMP7, CPP2, and CPP4. Cells were treated with vehicle (DMSO) and 50 µM of each peptide for 72 h. Results are representative of three independent experiments.
