# Supplementary material for: New Perspective for Using Antimicrobial and Cell-Penetrating Peptides to Increase Efficacy of Antineoplastic 5-FU in Cancer Cells
Source: J Funct Biomater. 2023 Dec 12;14(12):565. doi: 10.3390/jfb14120565 (PMC10744333; doi:10.3390/jfb14120565)
Supplement: Supplementary file 1 [file jfb-14-00565-s001.zip › jfb-2721996-supplementary.pdf]

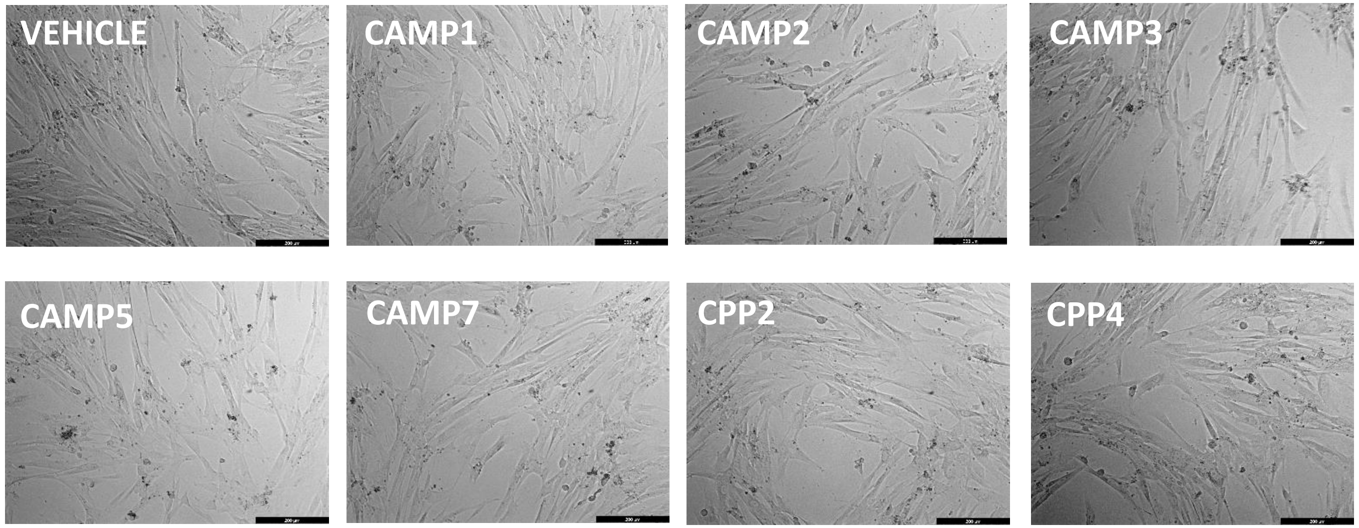

Figure S1. Morphological evaluation of MRC-5 cells treated with Peptides CAMP1, CAMP2, CAMP3, CAMP5, CAMP7, CPP2 and CPP4. Cells were treated with vehicle (DMSO) and 50 µM of each peptide for 72 h. Results are representative of three independent experiments. Scale bar: 200 µm.
